# Reaction-driven magmatic crystallisation at the Maoniuping carbonatite

**DOI:** 10.1038/s41467-025-62009-0

**Published:** 2025-08-04

**Authors:** Yan Liu, Michael Anenburg

**Affiliations:** 1 State Key Laboratory of Deep Earth and Mineral Exploration, Institute of Geology, Chinese Academy of Geological Sciences, Beijing, PR China; 2 Research School of Earth Sciences, Australian National University, Canberra, Australia

**Keywords:** Petrology, Mineralogy, Economic geology

## Abstract

Igneous rocks form by solidification of magmas through cooling or volatile degassing following decompression. Expelled H_2_O is thought to trigger alteration around intrusions, leading to formation of metasomatic halos. This mechanism is often invoked to explain many magmatic–hydrothermal rock associations, some of them economically mineralised. Maoniuping in China is one of the four largest operating rare earth element (REE) mines globally, whose origin has been attributed to such hydrothermal exsolution. However, no direct evidence links hydrothermal fluids to the formation of Maoniuping and its associated REE mineralisation. Here we show that the REE deposit at Maoniuping formed magmatically from a carbonatitic brine-melt. Textural and chemical evidence reveals extensive interaction with its quartz syenite host, producing albitised fenites. Coupled metasomatism with these fenites led to silica contamination of the carbonatite melt, triggering crystallisation of refractory alkali–ferromagnesian silicates—an antiskarn. This solidified the melt due to removal of the fluxing elements Na and K. Thus, carbonatite melts can crystallise by element assimilation from their environments, precipitating alkali liquid fluxes into solid minerals. Temperature decrease and volatile degassing merely play a secondary role in this igneous rock-forming process. Solidification driven by coupled antiskarnisation and fenitisation affects both the mineral assemblage and ore fabric, and likely operated in most carbonatite-hosted REE deposits elsewhere.

## Abstract (web summary)

Rare earth mineralisation at Maoniuping formed when its carrying carbonatite brine-melt reacted with surrounding siliceous rocks, forming an antiskarn. The melt lost its fluxing alkali elements, which led to deposition of coarse grained bastnäsite.

## Introduction

Most light REE (the rare earth elements La to Sm) are sourced from carbonatites, igneous rocks composed primarily of magmatic carbonate minerals such as calcite and dolomite^1–3^. The fundamental mechanisms that lead to the ubiquitous occurrence of REE deposits within these rocks are mostly understood^4^: Carbonatite rocks contain late-stage magmatic zones with Mg–Fe-rich carbonate assemblages of dolomite, ankerite, and siderite. These zones contain high concentrations of REE, which are typically incompatible in crystallising minerals during earlier stages of carbonatite melt differentiation (primarily calcite)^5,6^. Evolved carbonatite melts, now depleted of much of their initial CaCO_3_, resemble solute-rich brines and are often referred to as brine-melts to differentiate them from aqueous hydrothermal fluids as distinct coeval phases from these evolved carbonatite melts^4,7,8^. Fractionation of CaCO_3_ and MgCO_3_ leads to strong enrichment in other incompatible chemical components, most commonly H_2_O, Na, K, Ba, fluorine, sulfate, and, vitally, the REE^5,8,9^. This late stage, REE-rich, magmatic brine-melt then forms the mineralisation inside the dolomite–ankerite portion of the carbonatite^10^, often with associated voluminous barren calcite carbonatites surrounding it. Subsequent hydrothermal activity commonly redistributes REE from fluid-soluble primary igneous minerals (e.g., burbankite) to secondary alteration minerals (e.g., bastnäsite), and may remobilise the REE on a local scale of centimetres to several metres^4,11–13^. However, this remobilisation does not materially change the spatial distribution or grade of the REE (although it can change the mineral hosts, to the detriment or benefit to economic processing). Indeed, in carbonatite complexes where the entire calcite-to-ankerite sequence is observed, strong REE mineralisation is seldom found in calcite carbonatites, instead being overwhelmingly hosted by dolomite, ankerite, ferrocarbonatites, and their weathered products^13–16^.

Four carbonatite-associated REE deposits currently provide the majority of the world’s REE supply. Three of them contain substantial REE hosted by lithologies that fit into the aforementioned framework with REE mineralisation primarily hosted in iron-rich parts of carbonatites: The dolomite±calcite Sulphide Queen orebody at Mountain Pass (USA)^2,17^, the magnesio- and ferrocarbonatites at Mount Weld (Australia)^13,18^, and the banded dolomite at the Eastern Orebody of Bayan Obo (China)^19–24^. However, the fourth (Maoniuping, China) is unusual because its bastnäsite-dominated REE mineralisation is associated with calcite, lacking any dolomite or ankerite^25^. Moreover, most ore veins contain less than 30% calcite, placing them outside the carbonatite definition of the IUGS nomenclature guide or community conventions^26,27^. This led previous workers to conclude that the Maoniuping ore is not hosted by a carbonatite per se, but rather formed as a local hydrothermal offshoot of various fluid-exsolving alkaline carbonatite or silicate melts^21,28–35^.

As with most carbonatites, many currently observed minerals at Maoniuping contain abundant fluid inclusions, which trap hydrothermal fluids present during mineralisation^30,31,33,35,36^ (i.e., a synmagmatic fluid, if these fluids coexisted with a carbonatite melt or brine-melt^3^). The Maoniuping fluid inclusions are mostly hosted in quartz and fluorite, and they are rich in alkali sulfates, which have been suggested to facilitate REE mobility and mineralisation in carbonatite systems^21,28,30,31,35,36^. However, most Maoniuping fluid inclusion studies have found no ore or notable REE contents inside these inclusions^28,33,36^. Previous suggestions that a fluid in Maoniuping carried substantial REE are based solely on a single fluorite-hosted fluid inclusion^31,37^. This is problematic because fluorite itself is REE-rich, such that distinguishing fluid-hosted and mineral-hosted REE is challenging. Gangue-hosted fluid inclusions in hydrothermal ore-forming systems elsewhere typically contain the ore itself. For example, chalcopyrite is a ubiquitous daughter mineral in quartz-hosted fluid inclusions found in porphyry copper deposits^38^. The lack of ore-bearing fluid inclusions at Maoniuping raises the question of whether an ore-forming fluid, as a distinct phase from the carbonatite melt or its evolved brine-melt equivalent, existed at all. Some of the confusion might arise from ambiguity regarding fluids in carbonatite systems. Whereas the distinction is clear in silicate magmas that solidify at roughly 600–700 °C and coexist with an exsolved H_2_O-dominated fluid phase, relationships between fluids, brines, and melts in carbonatites prove somewhat inscrutable^8,39^. Recent work^7,9^ revealed that high-temperature carbonatite melts undergo continuous evolution to a lower temperature brine-melt, with temperatures as low as 300 °C, all the while remaining immiscible from the true H_2_O-dominated fluid^5^. The REE remain in this low-temperature magmatic brine-melt as opposed to the coexisting hydrothermal fluid^9^. Additional experimental work demonstrated high REE solubility in concentrated alkaline brines (i.e., brine-melts) at these temperatures^40^. Preliminary results indicate that REE might partition to a hydrothermal fluid in subvolcanic conditions (while remembering the melt–fluid distinction in carbonatite systems is not clear cut)^41^, but Maoniuping formed at depths where REE have been demonstrated to partition away from hydrothermal fluids (2–2.4 kbar^28,30^), remaining in the magmatic brine-melt instead^7,9,42^. Nevertheless, the discovery that low-temperature brine-melts exist as liquid phases that continuously differentiate from high-temperature carbonatite melts, all while being distinct from aqueous hydrothermal fluids, is relatively new^3,4,8^. Consequently, previous studies have likely misclassified magmatic low-temperature brine-melt inclusions as hydrothermal fluid inclusions^43^. The distinction can be particularly elusive if both brine-melt and fluid phases are entrapped in the same inclusion. In this case, a REE–carbonate-rich brine-melt might be diluted with H_2_O, NaCl, and Na_2_SO_4_. This obscures the magmatic character of the brine-melt and leads to erroneous interpretations that a REE-bearing hydrothermal fluid phase have existed, with the spurious conclusion that a magmatically-exsolved fluid is crucial for mineralisation^44^. The distinction between melts, brine-melts, and fluids is not only a matter of nomenclature—it has important implications for understanding spatial REE mobility within carbonatite systems, the timing of formation, changes in REE mineral hosts during alteration, and ultimately our capacity for effective REE exploration and exploitation.

The concept of an ore-forming fluid is predicated on the assumption that any vein deposit within an igneous complex, but without a clear magmatic mineral assemblage, formed from magmatic-derived hydrothermal fluids exsolved out of a crystallising magma, either local or adjacent^45^. This assumption is largely true for silicate igneous complexes^38^, but remains unsubstantiated in carbonatite systems. The presence of a separate synmagmatic hydrothermal fluid phase in equilibrium with actively forming magmatic mineralisation does not necessarily mean the fluid had any REE carrying capacity. Taken together with the extreme immobility of REE in calcite+fluorite saturated fluids^46–48^ and the preferential partitioning of REE to melts rather than fluids^9,42^, exsolved-hydrothermal formation models for the mineralisation at Maoniuping are tenuous, and a magmatic model should be considered. Nonetheless, mineral textures and relationships in Maoniuping are perplexingly different to other magmatically mineralised carbonatites. Here, we examine textures and mineral compositions from the Dagudao mining camp of Maoniuping and suggest a magmatic solidification process to explain its formation.

## Results and discussion

The Dagudao open pit contains three main ore-bearing rock units, from bottom to top: (1) stockworks, (2) veinlets, and (3) coarse ore veins. These rock units have been described in detail elsewhere^25,29^. All rocks described here have been photographed and sampled from the top ore vein unit around coordinates 101°58'57“E–28°26'59“N.

### Textural relationships

**Fig. 1:**
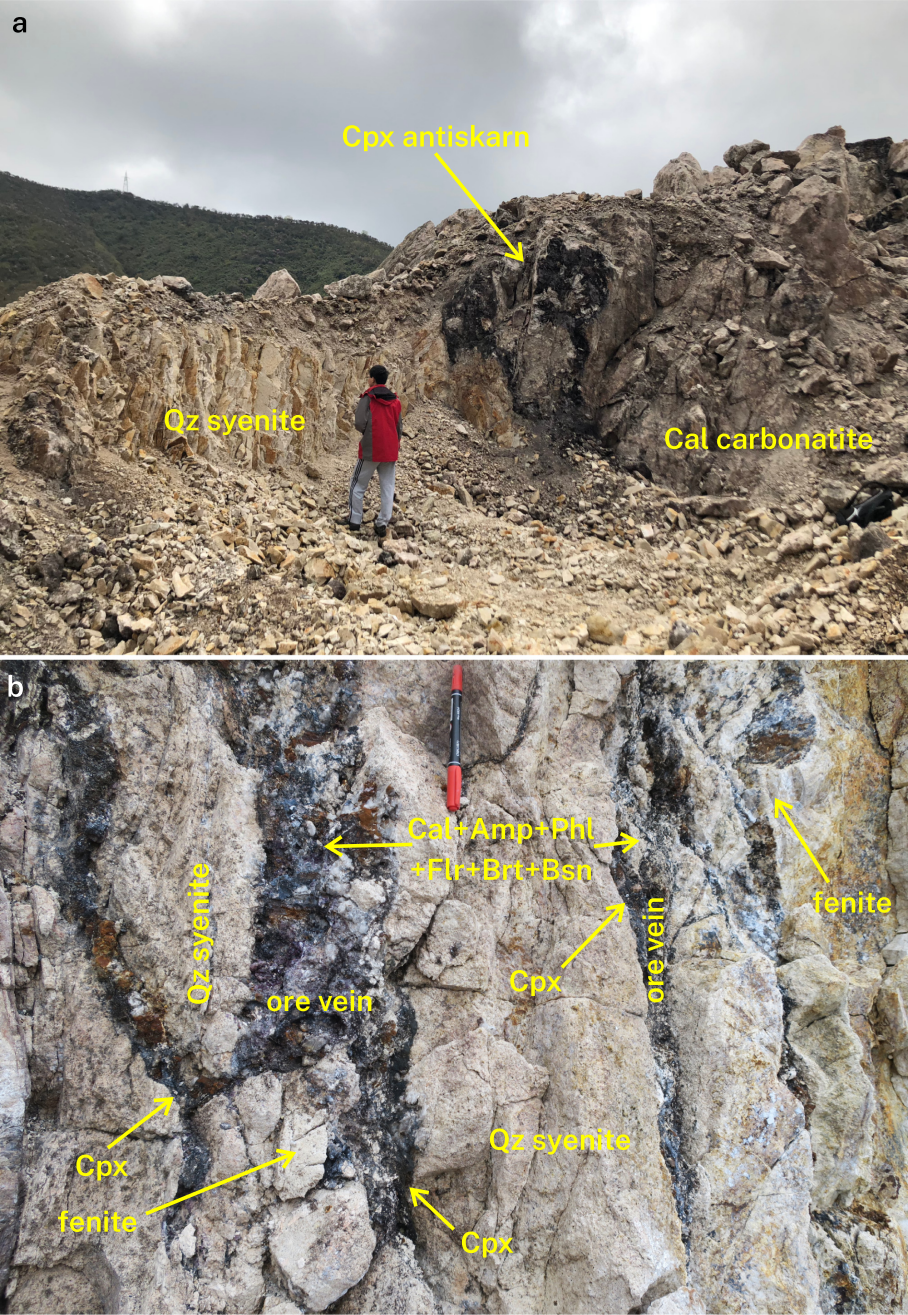
Field relationships between rock types at Dagudao, Maoniuping. **a** Rare example of a large calcite carbonatite body intruding into quartz syenite with a metre-scale pyroxene antiskarn at the contact. Individual ore veins are not visible at this scale, but are present in the outcrop in and around the antiskarn. Photograph taken in the main open pit, looking west. Similar calcite carbonatite bodies, often without substantial antiskarns and associated REE-mineralisation, are more common in the nearby Guangtoushan area, located about 500 m northeast of Dadugao. **b** Common bifurcating ore veins within quartz syenite. Most quartz syenites in this photograph have been albitised to fenite, with better-developed examples marked with an arrow. Abbreviations: Amp–amphibole, Brt–baryte, Bsn–bastnäsite, Cal–calcite, Cpx–clinopyroxene, Flr–fluorite, Phl–phlogopite, Qz–quartz.

**Fig. 2:**
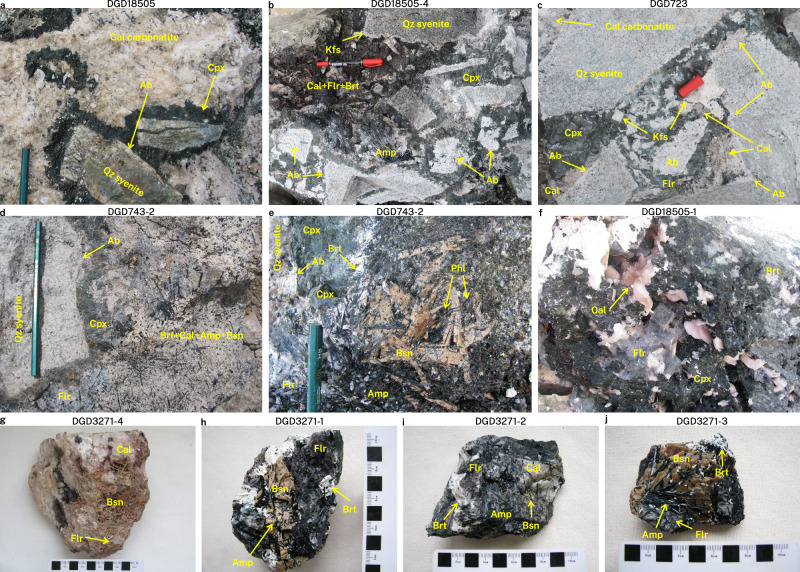
Mineral-scale features of Maoniuping rocks from the Dagudao mining camp. **a**–**d** Aegirine–pyroxene antiskarn reaction zones between quartz syenite and carbonatites with varying mineral proportions. **e**–**f** Pegmatitic growth into miarolitic cavities. **g**–**j** Common mineral associations and textures, always within centimetres of a pyroxene-dominated antiskarn. Sample names are annotated above the panels. Abbreviations: Ab–albite, Amp–amphibole, Brt–baryte, Bsn–bastnäsite, Cal–calcite, Cpx–clinopyroxene, Flr–fluorite, Kfs–K-feldspar, Phl–phlogopite, Qz–quartz.

**Fig. 3:**
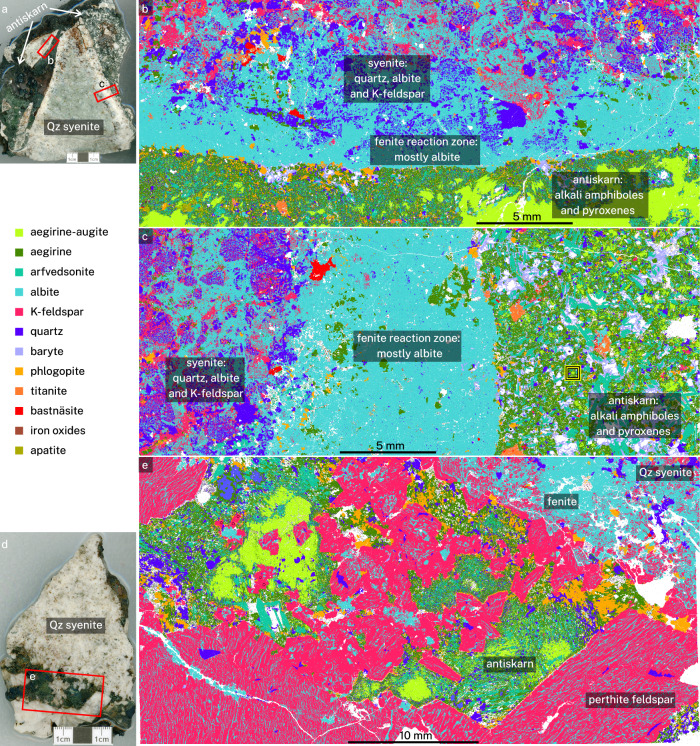
TIMA automated mineralogy images of bleached alteration rims (sample MNP14-4-3). **a** A quartz (Qz) syenite clast with red rectangles showing thin section locations. White areas are holes filled with epoxy resin. **b** Gradual decrease of quartz from syenite to antiskarn. Note abundant phlogopite at the contact. **c** Complete dissolution of quartz and K-feldspar from the syenite. Yellow–black square shows the location of pyroxene mapped in Fig. 4c–g. **d** A second syenite–antiskarn clast (sample MNP14-1) used for mapping. The red rectangle marks the approximate location of (**e**). **e** The antiskarn dominated by K-feldspar with perthite exsolution, zoned pyroxene, and abundant phlogopite along the fenitised quartz syenite. Samples obtained adjacent to ore veins similar to those shown in Fig. 2b, e.

Maoniuping comprises sub-vertical carbonatite and ore veins of various thicknesses, from the metre (Fig. 1a) to centimetre scale (Fig. 1b). These veins intrude into quartz syenite (often referred to as nordmarkite^28,34,49^) that forms the country rock. Pyroxene of aegirine-augite and aegirine compositions is ubiquitous (albeit not always present) at vein–syenite contacts in Dagudao (Fig. 2a–d and Supplementary Data 1). These pyroxene-rich zones contain minor amphibole, baryte, phlogopite, and K-feldspar (Fig. 3). Xenoliths of quartz syenite inside carbonatite retain their angular shapes and are coated by pyroxene overgrowths. This indicates that while vein interiors were liquid, pyroxene zones were forming outwards from the syenite contact, rather than replacing the syenite (Fig. 2a–d). These pyroxene coats are well-known from Maoniuping^25,32,35,36,50^. Quartz syenite rims at the contact with ore vein-hosted pyroxene are altered to bleached albite layers with almost no quartz (Figs. 2a–c, 3). The bleached alteration rim thickness roughly correlates with the thickness of the pyroxene layer that overgrows quartz syenite at the vein contacts. Phlogopite is occasionally concentrated in a thin layer between albite and pyroxene (Fig. 3). Large centimetre-scale K-feldspar crystals occasionally form in and around pyroxene layers, particularly where these layers are relatively thick (Fig. 2c).

Ore vein interiors consist primarily of baryte, fluorite, calcite, bastnäsite, phlogopite, and amphibole (Fig. 2e–j). Pyroxene also occasionally occurs in vein interiors. Quartz, when present, is always interstitial and likely to be secondary. The minerals are often coarse-grained and pegmatitic in appearance, and local metre-scale modal mineral compositions vary greatly (Figs. 1, 2). Calcite shows appreciable modal variability, ranging from > 90% to < 10%. This led previous researchers to define carbonatites sensu stricto, and other non-carbonatite ore veins, as two distinct lithological units on maps or cross sections of Maoniuping^51–53^. Despite this variability, calcite-dominated rocks are rare on the surface and more abundant in deep drill holes^25^. Both rock types—carbonatites and ore veins—are petrological cumulates (as distinct from mechanical cumulates), forming from carbonatite melts and differentiated brine-melts, respectively. Therefore, both liquids can react with surrounding silicate rocks and form antiskarn reaction zones (see below for more details).

**Fig. 4:**
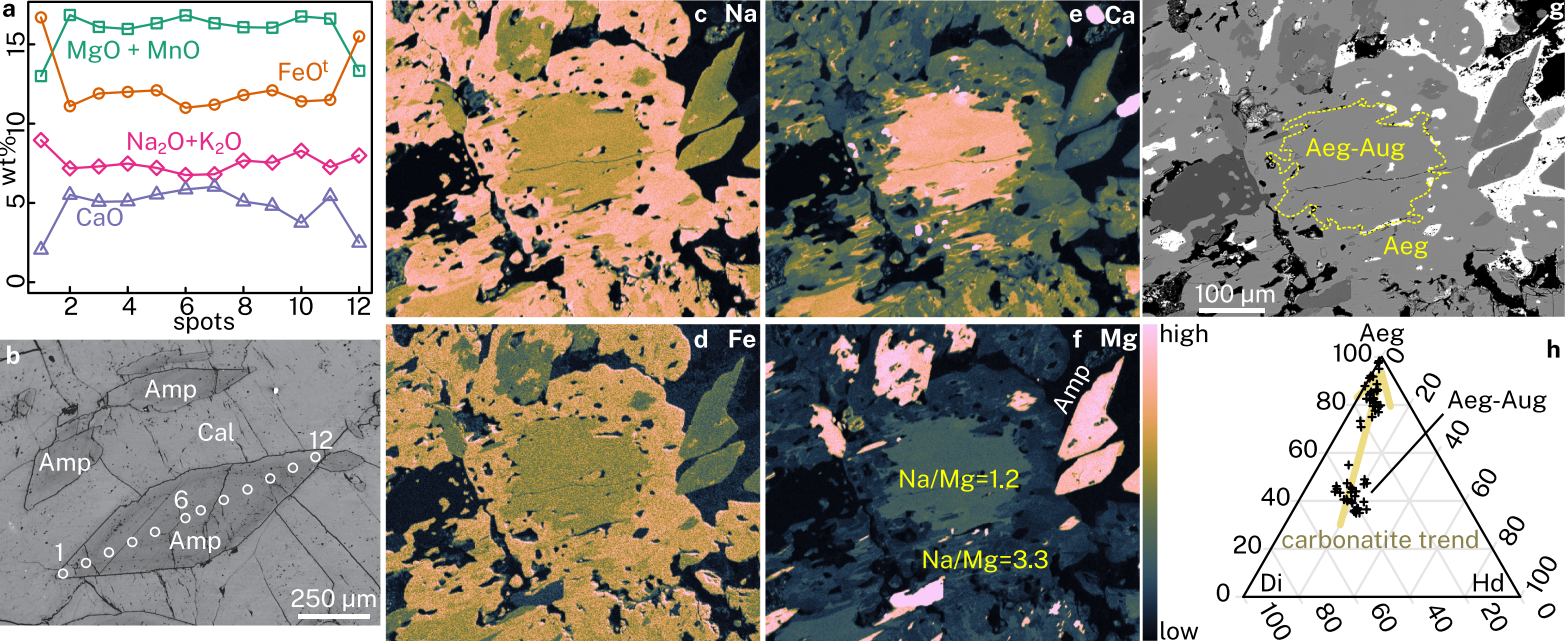
Mineral chemistry results at Maoniuping. **a** Chemical zoning profile of the amphibole shown in (**b**). **b** Backscattered electron image of a calcite-hosted amphibole, with numbered circles corresponding to analysis points in (**a**). **c**–**f** WDS elemental maps of pyroxene and amphibole, showing raw Na (**c**), Fe (**d**), Ca (**e**), and Mg (**f**) counts. **g** Backscattered electron image of pyroxene and amphibole mapped in (**c**–**f**). See yellow–black square in Fig. 3c for identification of associated minerals. The yellow outline in (**g**) shows two chemical zones (compositions shown in **f**) which are indistinguishable by BSE imaging. **h** Chemical compositions of all pyroxenes measured in this study projected on a diopside–hedenbergite–aegirine ternary diagram. Mineral compositions available in Supplementary Data 1. Abbreviations: Aeg–aegirine, Amp–amphibole, Aug–augite, Cal–calcite, Di–diopside, Hd–hedenbergite.

Veins at Maoniuping are commonly anastomosing without clear cross-cutting relationships (Fig. 1b). Instead, veins often bifurcate, indicating contemporaneous formation. Within veins, amphibole formed first, shown by its euhedral shape, inclusion in later minerals, and lack of truncation by other minerals (Figs. 2d, e, h–j, 4b). Apart from amphibole, no subsequent mineral crystallisation sequence is texturally apparent. Most calcite, baryte, fluorite, phlogopite, and bastnäsite crystals appear euhedral or nearly so, with occasional interpenetrating textures (Fig. 2e–j). Fractures inside one mineral are often filled with another, and vice versa, suggesting co-crystallisation. Crystal-lined cavities indicate open space crystallisation in miarolitic cavities (Fig. 2e, f).

### Mineral compositions

**Fig. 5:**
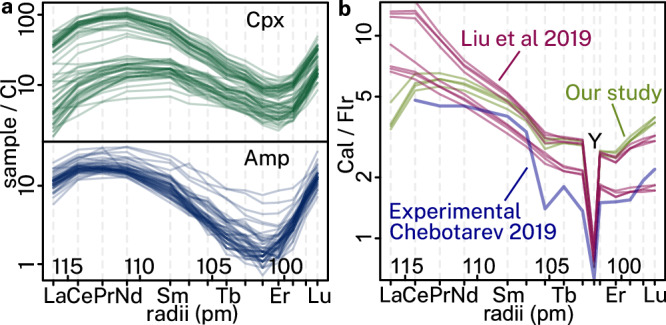
Trace element characteristics. **a** Chondrite-normalised (CI) rare earth element (REE) pattern for pyroxene (green, top) and amphibole (blue, bottom). **b** Calcite/fluorite REE partitioning patterns as determined experimentally (blue)^60^, and observed in this study from rocks similar to those shown in Fig. 2b, c, f, and g (green). See Supplementary Data 1 for sample locations and raw data, with additional data from the literature (violet)^29^. Abbreviations: Amp–amphibole, Cal–calcite, Cpx–clinopyroxene, Flr–fluorite.

Amphibole crystals are commonly zoned, with moderately sodic cores of richterite–ferro-richterite compositions, and highly sodic rims of arfvedsonite–magnesio-arfvedsonite compositions (Fig. 4a, b and Supplementary Data 1). Pyroxene compositions likewise show a trend from moderately sodic aegirine-augite cores to highly sodic aegirine rims (Fig. 4c–f, h and Supplementary Data 1). These zoning patterns are not readily apparent in BSE imaging and require elemental maps for characterisation (Fig. 4g). Pyroxene and amphibole compositional trends indicate that Na-bearing ferromagnesian silicate formation was characterised by increasing Na and Fe^3+^, concurrent with decreasing Ca and Mg contents^54^. Rare earth element patterns for both pyroxene and amphibole show a clear sinusoidal pattern with two inflection points, one at Pr–Nd, the other at Tm–Er (Fig. 5a). There is no substantial Eu anomaly. Similar patterns were reported before, demonstrating their widespread presence at Maoniuping^34^. These patterns are characteristic of igneous pyroxene and amphibole crystallised directly from carbonatite melts^55^, or of igneous pyroxene and amphibole that crystallised within silicate rocks in thermodynamic equilibrium with a carbonatite melt^56,57^, where high CaO/MgO activity ratios are implicated in triggering sinusoidality^58^. They differ from REE patterns of pyroxene and amphibole typical to other silicate rocks, in which pyroxene is often heavy REE-enriched, whereas amphibole is middle REE-enriched relative to both light REE and heavy REE.

Partitioning of REE between calcite and fluorite is also indicative of crystallisation from a carbonatite melt. Often, fluorite forms by neutralisation of acidic fluids, boiling of highly saline fluids, or fluid mixing of contrasting compositions. In all such cases, fluorite is unlikely to form in equilibrium with calcite. Specifically for carbonatites, hydrothermal fluorite forms by replacement of calcite (e.g., Okorusu, Namibia^59^). In contrast, the calcite–fluorite assemblages at Maoniuping appear to be in equilibrium. A pattern of calcite and fluorite REE partitioning in an experimental carbonatite magma system^60^ is given in Fig. 5b. REE ratio patterns observed in some calcite–fluorite pairs from Maoniuping are essentially identical (within a factor of up to two) to the experimental pattern (Fig. 5b). The order of magnitude difference probably results from the Maoniuping crystals forming at a lower temperature (< 700 °C, see below) than the experimental runs (710 °C^60^), or from different activities of chemical components that permit REE substitution in calcite and fluorite, such as monovalent charge-balancing cations^61^. Nevertheless, the parallel patterns indicate simultaneous equilibrium crystallisation of calcite and fluorite. There is no other plausible mechanism to generate calcite/fluorite REE ratios this close to experimental partitioning. Neutralisation of acidic fluids cannot exist in a carbonatite system because a calcite-saturated system could not exsolve acidic fluids to begin with, and other forms of hydrothermal fluorite deposition often require high NaCl or CaCl_2_ contents^62^, which were not observed in any Maoniuping fluid inclusion studies. Whilst fluorite and calcite REE patterns at Maoniuping vary^63^, these equilibrium pairs establish an evolutionary stage in which both co-crystallised, while later light REE-depleted fluorites^64,65^ record a syn- or post-solidification time in which most light REE were sequestered in bastnäsite.

### Maoniuping ore veins as magmatic carbonatites

The above results indicate that textural and chemical observations of shallow mineralised zones at the Dagudao mining camp of Maoniuping are consistent with magmatic rather than hydrothermal formation. Recent discussion of unusually low-temperature carbonatite melts such as brine-melts, and silicate–carbonatite reaction antiskarn processes^3^, may better explain Maoniuping. Recent drilling into Maoniuping revealed abundant unevolved and barren (relative to the upper ore veins) calcite carbonatites at depth^25^, supporting the view that early calcite fractionation is key, and any magmas forming the currently observed REE deposit on the surface had a strongly evolved brine-melt character. Nonetheless, both are of the same continuous magmatic differentiation series, consistent with previous Sr–Nd–Pb–C isotope studies showing a match between ore veins and more obvious magmatic carbonatites at Maoniuping^66^.

### Crystallisation via contamination

Key observations at Maoniuping are the pyroxene reaction zones. These zones have previously been interpreted as a result of fenitisation^28,67^—a process in which carbonatite-derived alkali fluids metasomatise surrounding silicate rocks, forming an assemblage of alkali silicates (including the currently observed K-feldspar and alkali amphiboles)^68^. However, the pyroxene zones clearly show sharp contacts with the quartz syenites, with pyroxene growth proceeding away from contact zones into veins (Figs. 2, 3). This indicates that the pyroxene zones crystallised inside the carbonatite melt conduit, and did not replace quartz syenite. Pyroxene preferentially nucleated at the syenite–carbonatite melt interface, but did not replace the syenite or any other rock. As such, the pyroxene zones are not fenites. Instead, these textures are identical to antiskarns revealed by experimental studies in which silica-free carbonatite melts reacted with solid silicate rocks^46^, and to natural systems where similar carbonatite–silicate reactions have been documented^58,69–73^. Alternatively, the Maoniuping pyroxene zones have previously been suggested to form by simple fractionation of silica-bearing carbonatite magmas^74^. However, as carbonatite melts below about 1000 °C contain negligible dissolved silica^75–78^, this is unlikely. Instead, we propose that silica was supplied by the host quartz syenite, supported by the bleached zones, which essentially consist of the original mineral assemblage of the quartz syenite, minus quartz and K-feldspar (as observed elsewhere^10,71^).

Whereas pyroxene was experimentally shown to form at the contact itself, other ferromagnesian minerals form farther inside the carbonatite melt^46^. In addition, occasional layers of phlogopite on the syenite–antiskarn contact (Fig. 3b) are consistent with previous studies of silica contamination in carbonatites^10,70,79^, with SiO_2_ and Al_2_O_3_ sourced from quartz syenite xenoliths, MgO from the carbonatite melt, and K_2_O from both, producing decreased K-feldspar contents in the albitised zones. Formation reactions of early pyroxene can be written considering the Ca–Mg endmember

1 $$({{\rm{melt}}}) \qquad \, ({{\rm{rock}}}) \quad ({{\rm{diopside}}})\quad\,({{\rm{gas}}})\,\, \\ {{\rm{CaMg}}}({{{\rm{CO}}}}_{3})_2+2{{\rm{Si}}}{{{\rm{O}}}}_{2}={{{\rm{CaMgSi}}}}_{2}{{{\rm{O}}}}_{6}+{2{{\rm{CO}}}}_{2}$$

and formation reactions of late pyroxene can be written considering the Na–Fe^3+^ endmember

2 $$({{\rm{melt}}}) \quad \,({{\rm{melt}}})\quad ({{\rm{rock}}})\,\quad ({{\rm{aegirine}}})\quad \,({{\rm{gas}}})\\ {{{\rm{Na}}}}_{2}{{{\rm{CO}}}}_{3}+{{{\rm{Fe}}}}_{2}{{{\rm{O}}}}_{3}+{4{{\rm{SiO}}}}_{2}={2{{\rm{NaFeSi}}}}_{2}{{{\rm{O}}}}_{6}+{{{\rm{CO}}}}_{2}$$

The calcic cores and sodic rims of pyroxene (Fig. 3h) and amphibole are consistent with thermodynamic modelling of silica contamination progression of a carbonatite melt^54^, and are consistent with the compositional range of natural magmatic carbonatite-hosted pyroxene^55,80,81^. Importantly, reaction 2 consumes Fe^3+^, consistent with the lack of magnetite at Maoniuping, a mineral typical of calcite carbonatites elsewhere. Carbonatites crystallising from brine-melts, such as dolomite and ankerite carbonatites, are expected to have very little to no magnetite, since Fe^3+^ has been fractionated away. As the Maoniuping ore veins likely formed from such a brine-melt, Fe^3+^ was present in very small amounts, or not at all. In the absence of available Fe^3+^ within the carbonatite melt, Fe^3+^ can be generated by reduction of CO_2_ to methane^54,82^:

3 $$\begin{array}{c}({{\rm{melt}}})\qquad({{\rm{melt}}})\,\qquad({{\rm{rock}}})\,\quad({{\rm{melt}}})\qquad ({{\rm{aegirine}}})\,\,\,\,\,\quad ({{\rm{gas}}})\quad ({{\rm{gas}}})\\ {4{{\rm{Na}}}}_{2}{{{\rm{CO}}}}_{3}+{8{{\rm{Fe}}}}^{2+}{{{\rm{CO}}}}_{3}+{16{{\rm{SiO}}}}_{2}+{2{{\rm{H}}}}_{2}{{\rm{O}}}={8{{\rm{NaFe}}}}^{3+}{{{\rm{Si}}}_{2}{{\rm{O}}}}_{6}+{{{\rm{CH}}}}_{4}+{11{{\rm{CO}}}}_{2}\end{array}$$

Evidence for this process is gleaned from fluid inclusion studies of Maoniuping reporting enigmatic CH_4_-bearing carbonic inclusions^30^, which would otherwise be unexpected in a typically oxidised, sulfate-rich, carbonatite system^83^. Similar reactions can be written for amphibole, and when considering K_2_O in syenite or K_2_CO_3_ in the carbonatite melt, also for phlogopite^54^. This sequestration of Mg and Fe in ferromagnesian silicates prevents the formation of dolomite and ankerite, leaving calcite as the only carbonate mineral forming from the melt^54,79,84^.

The tabular K-feldspar grains forming in the antiskarn, adjacent to the syenite xenoliths (Figs. 2c, 3e), provide additional evidence for silica transport into the carbonatite melt. Based on previous thermodynamic modelling, two distinct feldspars indicate typical formation temperatures of up to about 650 °C^54^. Rare alkali feldspar perthites (Fig. 2) concordantly suggest the process started at < 700 °C^54^, since higher temperatures would stabilise nepheline^73^ or kalsilite, which are not observed at Maoniuping. This temperature range agrees with published melt inclusion studies^30^. Antiskarn formation and associated decarbonation reactions are highly pressure dependent^54^, with silicate–carbonatite disequilibrium preferred at shallower settings. This is consistent with the abundant antiskarn textures observed near the surface and described above. In contrast, evidence for these reactions is much less common in rocks obtained from drill holes that reached almost 1000 metres depth^25^. Similar textures were observed elsewhere in the Mianning-Dechang belt, including at Muluozhai^44^ and Dalucao^34^.

### Fenitisation–antiskarnisation by reaction with carbonatite melts

Fenites are traditionally understood to form via aqueous alkali-rich fluids expelled from carbonatite melts^68^. In contrast, here fenites form directly by reaction with the carbonatite melt or its more evolved derivative brine-melt with no involvement of any aqueous fluids. That is, the carbonatite melt itself is the metasomatising agent. Formation of fenites occurs alongside the transfer of silica from the silicate rocks to the carbonatite melt, in which refractory silicate minerals form. These feldspar-dominated reaction rims formed by replacement of the original quartz syenite after extraction of silica by carbonatite melts; therefore, they are classified as fenites (whereas pyroxene does not, in fact, belong to the fenites). In this case, the formation of ferromagnesian silicates as part of the antiskarn assemblage and the albitisation of the host silicate rocks to fenites is a coupled reaction, and one cannot happen without the other.

We have no reservations regarding fenites forming via alkaline aqueous fluids, which undoubtedly exist as a separate liquid phase in carbonatite systems. However, we suggest that many traditionally recognised fenites may actually be antiskarn–fenite pairs in which the carbonatite melt has been completely reacted out, resulting in no carbonate minerals that indicate the former existence of this melt.

### Metasomatic loss of flux elements and solidification of the residual brine-melt

It is well known that Na and K are efficient fluxing elements that strongly reduce the solidus of carbonatite melts, demonstrated by both experimental studies^85^ and natural observations from the only erupting natrocarbonatite at the Oldoinyo Lengai volcano in Tanzania^86^. Melt inclusion studies of Maoniuping show an upper limit of about 850 °C^30,36,87^. Most of these inclusions were observed in quartz, but quartz is not an equilibrium high-temperature phase in carbonatite melts. Quartz can only be stable in a carbonatite system at temperatures below the calcite + quartz = wollastonite curve at about 500–600 °C (depending on pressure and CO_2_ activity)^54^. This indicates that quartz melt inclusion contents have been made refractory by the antiskarn-forming reactions, leading to spuriously high apparent trapping temperatures^8^. For a carbonatitic brine-melt to be liquid at 600 °C and lower, it requires abundant Na or K^5,8,85^.

An inevitable consequence of alkali-rich ferromagnesian mineral formation is the removal of Na and K from the carbonatite melt. A 10% decrease in alkali carbonate components is equivalent to a ~ 100 °C increase in the carbonatite melting point^85^. Once alkalis are progressively sequestered in silicate minerals, fewer fluxes remain to maintain the liquid state, and this process advances crystallisation of all other components, some of them also acting as fluxes, notably with baryte sequestering sulfate. This process likely occurs in a non-isothermal environment, since hot brine-melts are encountering presumably cooler host rocks, excess vapour is advecting heat upwards, and mineral formation releases latent heat of crystallisation. However, any temperature variations are negligible in this solidification mechanism. When considering the driving forces for known magmatic solidification processes in temperature–pressure–composition space, most systems are explained by either temperature decrease or decompression-led volatile loss. Here, the cause for solidification is composition change. Any changes in temperature or pressure, although likely to occur, do not play an important role.

The formation of the silicate antiskarn assemblage is accompanied by the generation of CO_2_ gas derived from decarbonation reactions (e.g., Reactions 1, 2 and 3). Carbon isotopes of calcite from Maoniuping record this decarbonation process^25^. Alkali consumption to silicates also removes cations from alkali sulfates, leading to degassing of SO_2_ (evident by previous S isotope studies^50,88^). Crucially, ore-forming chemical components are primarily carried by the magmatic brine-melt, and not dilute gas nor any hydrothermal fluids^8,42^. Gas release is also likely responsible for the breccia textures observed at Maoniuping (e.g., Fig. 2a–d), as has been demonstrated elsewhere for antiskarn-bearing subvolcanic carbonatite systems^89^. Once solidification is complete, cooling occurs, and barren residual hydrothermal fluids are eventually captured as fluid inclusions^39^, primarily in late-stage, post-mineralisation, hydrothermal quartz. We also note that many fluid inclusions are observed in the readily cleaved fluorite and bastnäsite. In the violent environment of brecciation during antiskarn formation, many crystals can fracture with late fluid inclusions trapped inside cleavage planes. At Maoniuping, this is demonstrated by observation of, among other textures, sheared amphibole, bent calcite, and fractured fluorite and bastnäsite with baryte and quartz infilling, respectively^25^. After healing, they can be easily mistaken for primary inclusions trapped during crystal growth. The character of these fluid inclusions contributed to the erroneous interpretation that Maoniuping formed hydrothermally. The fluid inclusions often contain soluble alkali sulfates and chlorides, but lack some of the crucial chemical components that make up Maoniuping’s solid mineral assemblage: Ba in baryte, F in fluorite, and the REE. These elements are only substantially soluble and mobile in acidic fluids^47,48^, but such low-pH fluids are not attainable in a calcite-bearing carbonatite system, as they could not form in the first place, and they will be immediately neutralised if somehow formed or existed. This Ba-F-REE immobility indicates that all mineralisation formed magmatically, and later hydrothermal fluids were unreactive with the already solidified mineral assemblage.

### Preservation of soluble brine-melts

A major challenge with the study of brine-melts lies in their ephemerality, since they are dominated by soluble salts and not preserved in nature. An instructive example is found in the natrocarbonatite lavas of Oldoinyo Lengai, which dissolve and alter within hours after eruption, leaving almost no record of their existence for preservation in geological history^90,91^. Likewise, experimental studies that reached evolved carbonatite melt compositions and brine-melts show that they are dominated by soluble components, and require special preparation and analytical techniques even in a laboratory setting^5,7,9,85,92^. Thus, any expectation for solidified brine-melts to be preserved in the geological record is futile^93,94^. This seriously hampers the development of magmatic models for carbonatite formation and associated REE mineralisation. Conversely, solidified silicate magmas are invariably retained in the geological record. Other than haphazardly trapped melt inclusions, for which interpretation is often contentious, there is no clear evidence for the former existence of brine-melts in most carbonatites^8^. This leads to the widespread opinion that hydrothermal fluids must have been implicated in ore formation. Here, Maoniuping provides a unique opportunity to observe preserved brine-melts in the form of refractory silicate minerals.

The zoning from Ca–Mg-dominated amphiboles and pyroxenes to their sodic equivalent traces brine-melt formation. In a non-contaminated system, a brine-melt forms after fractionation of calcite and dolomite^3^. As CaCO_3_ and MgCO_3_ are the most abundant components of primitive carbonatite melts, their removal requires enrichment of all other components. Thus, formation of a brine-melt is inevitable as a carbonatite melt differentiates^4,5^. At Maoniuping, although abundant calcite cumulates are present in deep drilling and occasional surface exposure (Fig. 1a), no dolomite cumulates are present, and the presumably inevitable formation of a brine-melt is not attained. However, the removal of CaCO_3_ and MgCO_3_ as calcite and dolomite is only one way to achieve this differentiation. Alternatively, in silica-contaminated systems, these components can be sequestered to refractory silicates such as amphibole and pyroxene and removed from the carbonatite melt^85^, with the excess CO_2_ degassed instead of forming carbonates. In this way, the inevitable brine-melt formation is realised, and recorded by the continuous zoning from the initial Ca–Mg silicate cores to their alkaline equivalents at their rims.

In non-contaminated systems, brine-melts crystallise to an assortment of non-silicate minerals^4^. Alkali carbonates such as nyerereite and gregoryite are the primary hosts of Na and K, while REE and Ba form burbankite and carbocernaite^95,96^. Dolomite, ankerite, magnesite, and siderite often sequester most Mg and Fe. A variety of other alkaline minerals host additional anions such as fluoride, sulfate, and phosphate. For instance, experiments revealed the formation of eitelite, bonshtedtite, moraskoite, rouvilleite, neighborite, and parascandolaite^5^, with some of these or other related minerals observed in fluid or melt inclusions within natural carbonatite complexes^8,94,97–102^. These minerals are soluble, with the alkalis Na and K readily removed by later fluids. The preserved mineral assemblage is almost entirely devoid of alkalis^26^, with non-soluble elements redistributed to secondary carbonates or phosphates, leading to the false appearance of primary hydrothermal deposition^4^.

In Maoniuping, the non-silicate phase assemblage is not substantially different to the ubiquitous secondary assemblages found in most carbonatites, with abundant bastnäsite, baryte, and fluorite. However, their conspicuous phenocrystic morphology (Fig. 2) indicates primary formation instead of in situ replacement of soluble alkali-rich minerals. This texture was previously inferred as hydrothermal formation, but we suggest their presence is indicative of antiskarnisation. Silica contamination triggered the formation of alkali silicates (aegirine, phlogopite, and arfvedsonite)^54^, preventing Na and K from forming any soluble minerals and causing other elements (e.g., REE, Ba, F) to crystallise into insoluble minerals. These insoluble minerals (bastnäsite, baryte, and fluorite, respectively) are the same minerals that would have formed had soluble minerals (e.g., burbankite) magmatically crystallised in the first place, and then hydrothermally dissolved^9^. The difference is textural, with these minerals now appearing coarser and well-formed, and even pegmatitic in some cases. The alkalis themselves were retained in the system, sequestered in silicate minerals^89^. In essence, the externally introduced silica served as the glue which kept most components of the brine-melt within the system, instead of permitting them to be washed away as soluble carbonates and other minor salts^9^. Even components which were not strictly bonded to silica, such as carbonate, fluoride and sulfate, lost their alkali cation counterpart, which initially kept them in the melt. Instead, these components directly formed insoluble and well-faceted bastnäsite, fluorite, and baryte, respectively. Using REE as an example, bastnäsite is the principal ore mineral at Maoniuping, and it exhibits clear pegmatitic textures (Fig. 2e, g–j). The igneous formation of the Maoniuping bastnäsite stands in stark contrast to its typical occurrence in most carbonatites elsewhere, where bastnäsite forms as a late postmagmatic hydrothermal replacement of ephemeral alkali REE carbonates such as burbankite or carbocernaite^4,11,12^.

Experimental work demonstrated that magmatic bastnäsite does not form in alkali-rich carbonatite melts^96^. Instead, it can only form when no alkali elements are present in the melt^5^, when the melt reaches exceptionally high contents of dissolved bastnäsite^95^. In nature, bastnäsite typically forms by dissolution of the Na_2_CO_3_ component in burbankite. At Maoniuping, bastnäsite formation cannot be attributed to hydrothermal burbankite replacement, as alkalis were sequestered in ferromagnesian silicates. This prevented burbankite from crystallising in the first place. This explains the previously-suggested magmatic origin for bastnäsite^33^ and further supports our genetic model in which alkalis are removed to refractory silicates (Fig. 4a, b), followed by subsequent solidification of the residual melt in an actively alkali-depleting melt system.

### Assimilation-induced crystallisation of carbonatitic brine-melts

Maoniuping is relatively young having formed during the Oligocene^33,49,66^. It is spectacularly preserved with deep, unweathered sections exposed due to active quarrying. The Maoniuping veins are long and thin, allowing ample contact with the host quartz syenites. Therefore, the formation of its shallow REE-rich portion was entirely dominated by the antiskarn-forming reactions and resulting reaction-driven solidification, and it serves as the type locality for this endmember process. In both surface exposure and deep drilling, the lack of surrounding distal fenites (indicating a lack of excess Na and K) and lack of dolomite or ankerite (indicating a lack of available Mg and Fe) patently demonstrate that alkali-consuming reactions progressed to completion. In contrast, other carbonatite complexes often appear as semi-circular ring intrusions, with sizes from many tens to a few hundred of metres across. In these circular complexes, reaction-driven solidification would only occur at the edges, close to the contact with the siliceous country rocks. Formation of aegirine and other silicates would then put a barrier, armouring the internal carbonatite melt from reacting any further and permitting the residual melt to fractionate according to the predictable dolomite and ankerite evolutionary path^103^. The outer rings of many carbonatite complexes are dominated by silicate minerals with ubiquitous pyroxene of carbonatite trend compositions (e.g., Fig. 4h). These silicate rings were previously explained as the products of silicate-carbonatite melt immiscibility, or alternatively as early fractionated cumulates from a single carbonated silicate melt^57,104^. However, these silicate-rich zones of carbonatite complexes occasionally contain REE carbonates^57^, or Ba-Sr minerals^56^, which would be exceptionally unusual in early-stage rocks, because these are some of the most soluble elements in carbonatite melts and only become substantially enriched in late-stage brine-melts^8,95,105^. Their characteristic niobium enrichment also supports reaction-driven formation and antiskarnisation^106^.

The world’s largest deposit, Bayan Obo, primarily contains REE in the so-called banded dolomite ores, but contains additional substantial mineralisation within calcite carbonatite dykes (Wu dykes^107^) surrounded by silicate rocks containing mostly aegirine and riebeckite (an alkali amphibole), occasionally in contact with quartz conglomerate country rocks. In these calcite carbonatite dykes, the mineralisation often occurs as phenocrystic grains of bastnäsite, parisite and monazite, with associated baryte and fluorite^107–109^. Although previously interpreted as fenites, the similarity to Maoniuping is striking, such that a likely interpretation is that the silicate-rich rocks are antiskarns, and the mineralised calcite carbonatites are metasomatically contaminated alkaline carbonatite brine-melts. Although a clear separation between antiskarns (ferromagnesian minerals) and fenites (albitised silicate host rock) is not yet clearly reported from Bayan Obo^109^, albite is an abundant mineral in the reaction zones^110^, and we predict that further investigation will reveal increasing albite contents towards the host silicate rocks. Likewise, Mountain Pass contains several calcite carbonatite dykes with bastnäsite and baryte phenocrysts, which intrude into surrounding siliceous country rocks very much like observed in Maoniuping^2,17^.

The exact mineral assemblage composition of the antiskarn–fenite pair need not necessarily be like in Maoniuping. Here, the carbonatite was mostly sodic and intruded into SiO_2_ and Al_2_O_3_ rich quartz syenite. This manifested itself as albite in fenite and aegirine in the juxtaposed antiskarn. Other cases might be different. For example, a more potassic carbonatite intruding into pelitic sediments or their metamorphic equivalent might result in abundant phlogopite or biotite (as often occurs around many carbonatites, with glimmerites in Mount Weld being a notable example^13^). Even in the deeper sections of Maoniuping itself, one can find small antiskarn–fenite pairs that represent a more potassic composition with K-feldspar and phlogopite as the main products, in contrast to albite and aegirine in the shallower sections shown here^25^. Equivalent mica-dominated reaction zones are observed elsewhere in the Mianning-Dechang belt (e.g., Lizhuang and Dalucao^34^). Similarly, carbonatite melts of varying compositions intruding an even greater chemical variety of host rocks can lead to a plethora of fenitisation and antiskarnisation styles. Focus should be put on the mechanism of silica transfer and alkali sequestration, rather than the presence of any individual mineral (such as albite, phlogopite or aegirine).

REE deposits typically form in the shallower levels of mostly vertically-zoned carbonatite intrusions^111^. It is in these depths and low pressures that antiskarn formation reactions are favoured^54^. Given the diversity of near-surface host rocks, we propose a crucial role for their composition—and particularly their silica contents—that control mineralisation style in the carbonatites that intrude into them. We suggest that all carbonatite-hosted REE deposits form along a continuum between negligible interaction with the country rocks (e.g., Kankgankunde, Chilwa Island^15^ and Saint-Honoré^14^), where the country rocks are silica poor and rarely lead to antiskarn formation, to complete interaction (the REE deposit at Maoniuping). The location of a single carbonatite complex along the continuum has important implications for its hosted REE mineralisation. Ideally, the economics of a deposit are improved when the ore is concentrated in a small volume. As reaction-driven solidification extracts REE from the melt into the solid phase, limited antiskarn formation (such as in ring complexes) will deplete some REE from the carbonatite melt, leaving less ore of value in the residual melt, which would then fractionate to non-ideally mineralised ferrocarbonatites. In this case, the total REE budget of the carbonatite system would be detrimentally spread over the large volume of both the early calcite carbonatites and later ferrocarbonatites. Alternatively, when the carbonatite melt intrudes as thin veins with abundant reaction surfaces (as in Maoniuping^25^), the REE are deposited within the antiskarn in their entirety, because the evolved carbonatite and brine-melts are completely consumed and never advance to ferrocarbonatite formation. The above discussion demonstrates that ideal REE concentration would occur at the two ends of the continuum: either full reaction or none, whereas partial reaction would act to lower REE grades.

## Methods

### Electron microscopy and analysis

Back-scattered electron (BSE) images and major-element analyses of minerals were acquired using a JXA-8230 electron probe microanalyser (EPMA) at the Institute of Mineral Resources, Chinese Academy of Geological Sciences (CAGS), in Beijing, China. The EPMA was operated at an accelerating voltage of 15 kV, a beam current of 20 nA, and a beam size of 5 μm. The following natural and synthetic standards, analytical lines and detector types were used for EPMA analysis: jadeite (Na-Kα and Al-Kα on TAP, Si-Kα on PET); forsterite (Mg-Kα on TAP); topaz (F-Kα on TAP); K-feldspar (K-Kα on PET); wollastonite (Ca-Kα on PET), hematite (Fe-Kα on LIF); rutile (Ti-Kα on LIF); MnO (Mn-Kα on LIF); NaCl (Cl-Kα on PET). Matrix corrections were carried out using the ZAF correction programme supplied by the manufacturer. Element maps were acquired using a beam current of 100 nA, a beam size and pixel spacing of 1 µm, and 50 ms dwell time per pixel.

Mineral maps were obtained using a TESCAN Integrated Mineral Analyser (TIMA) system at the Xi’an Kuangpu Geological Exploration Technology Co., Ltd. The TIMA system comprises a TESCAN MIRA3 Schottky field emission SEM and four high flux EDS detectors (EDAX Element 30) arranged at 90° intervals around the chamber. We used a spatial resolution of 9 μm and 1200 X-ray counts per pixel. Operating conditions were an acceleration voltage of 25 kV and a beam current of 9 nA. Measured BSE and EDS data were matched with a phase database for rapid mineral identification.

### In situ trace element analysis

In situ trace element compositions of both pyroxene and amphibole were determined by an excimer 193 nm ArF Analyte Excite Laser ablation inductively coupled plasma–mass spectrometry (ICP-MS) system, coupled to an Agilent 7700x at the Nanjing FocuMS Technology Co. Ltd., Nanjing. This was carried out on the same spots which had been analysed by EPMA and analysed trace elements include Rb, Sr, Ba, Th, U, Nb, Ta, Zr, La, Ce, Pr, Nd, Sm, Eu, Gd, Tb, Dy, Y, Ho, Er, Tm, Yb, Lu, Hf. The analyses condition involved a 7 Hz repetition rate and a beam diameter of 25−40 μm. In addition, BCR-2, BHVO-2, AVG-2, and RGM-2 glasses were used as external calibration standards, and Chinese Geological Standard Glasses (CGSG)-1, -2, -4, and -5 (prepared by National Research Centre for Geoanalysis, Beijing, China) were treated as quality control. Raw data reduction was performed offline by ICPMSDataCal using 100% normalisation, without applying an internal standard.

### Bulk trace element analysis

Representative mineral samples were prepared using conventional crushing, sieving, and heavy liquid separation. Trace element analyses of both calcite and fluorite were performed at the National Research Centre of Geoanalysis, CAGS, Beijing, China. For analyses of trace elements and REEs, whole-rock powder samples (50 mg) were dissolved in distilled 1 mL HF and 0.5 mL HNO_3_ in 15 mL Savillex Teflon screw-cap capsules at 190 °C for one day, dried, digested with 0.5 mL HNO_3_, and then dried again. The capsule content was digested with 0.5 mL HNO_3_ and dried again to ensure complete digestion. Then, the sample was digested with 5 mL HNO_3_ and sealed at 130 °C in an oven for 3 h. After cooling, the solution was transferred to a plastic bottle and diluted to 50 ml before analysis. The sample solutions were analysed for trace elements by ICP-MS. According to the procedure of State Standard of the Peoples Republic of China (GB): Methods for chemical analysis of silicate rocks-Part 30: Determination of 44 elements. The analytical precision for most elements was better than 5% according to the lab work procedure, repeatability and reproducibility of standard samples measurement results. To verify the procedure’s accuracy and precision, several standard samples of GBW07120, GBW 07103, GBW 07105 and GBW 07187 were analysed together with other samples.

## Supplementary information

Description of Additional Supplementary Files

Supplementary Data 1

Transparent Peer Review file

## Data Availability

All data supporting the findings in this paper are available in the supplementary information files.
