# Supplementary material for: Reaction-driven magmatic crystallisation at the Maoniuping carbonatite
Source: Nat Commun. 2025 Aug 4;16:7159. doi: 10.1038/s41467-025-62009-0 (PMC12322019; doi:10.1038/s41467-025-62009-0)
Supplement: Supplementary file 1 — Description of Additional Supplementary Files [file 41467_2025_62009_MOESM1_ESM.pdf]

Description of Additional Supplementary Files:

Supplementary Data 1: Tables in Excel format containing major and minor trace element compositions for pyroxene and amphibole, trace element compositions for calcite and fluorite mineral pairs, and major element data for spots in mapped pyroxene crystal.
