# Supplementary material for: Reaction-driven magmatic crystallisation at the Maoniuping carbonatite
Source: Nat Commun. 2025 Aug 4;16:7159. doi: 10.1038/s41467-025-62009-0 (PMC12322019; doi:10.1038/s41467-025-62009-0)
Supplement: Supplementary file 3 — Transparent Peer Review file [file 41467_2025_62009_MOESM3_ESM.pdf]

nature portfolio

Peer Review File

Reaction-driven magmatic crystallisation at the Maoniuping carbonatite

Corresponding Author: Dr Michael Anenburg

This manuscript has been previously reviewed at another journal. This document only contains information relating to versions considered at Nature Communications.

This file contains all reviewer reports in order by version, followed by all author rebuttals in order by version.

Version 1:

Reviewer comments:

Reviewer #1

(Remarks to the Author)
The submitted manuscript represents a strongly revised version of a manuscript, which I previously reviewed. Most of the concerns I raised back then (March 2022) are well addressed now and apart from few minor things I do not have any major objectives anymore. All of my comments are given in the attached manuscript file and I hope that they are of some use. I recommend publication of this manuscript after minor revisions.

Reviewer #2

(Remarks to the Author)
Review report on ‘Magma solidification via reaction freezing at the Maoniuping carbonatite’

Please note all line numbers are based on the annotated Word document opened on Mac. Minor changes may occur in a Window system.

(1) Significance and focus of this study
The goal of this study is to demonstrate that the pyroxene-amphibole zones (antiskarns) are linked to the vein-type REE mineralisation style at Maoniuping. Instead of a hydrothermal origin, the authors propose that the mineralisation formed magmatically from low-temperature brine-melts that metasomatized the country rock. While the formation of antiskarns via metasomatic exchanges between a brine-melt and the country rock seems reasonable, the (potential) genetic link between the antiskarns and the REE mineralisation has not been thoroughly investigated. The simultaneous antiskarn formation and ore-vein crystallisation should first be confirmed, before concluding that the two are inherently related. Additional geological/petrographic/geochemical evidence is required to support this hypothesis.

In addition, the authors suggest that the REE mineralisation formed by isothermal ‘’metasomatic freezing’’. This is a novel petrological process that, according to the authors, results in the isothermal solidification of a melt. Although the general mechanism (removal of alkali fluxes from the melt) is plausible, the thermodynamic evidence has not been provided. Primary crystallisation temperatures for the antiskarns and the REE ore-veins should also be determined to support this claim. Furthermore, the authors appear to contradict themselves on the isothermal nature of this process.

All in all, the authors address some important and relevant points that definitely deserve attention. However, the authors draw some major conclusions from rather limited lines of evidence, and particularly lack reliable geology information derived from their own study. For example, the ‘’entire formation’’ of Maoniuping has been suggested to be the result of ‘’antiskarn forming reactions and resulting reaction freezing’’, while only a few outcrops have been studied. The authors also state themselves that ‘’evidence for these reactions is much less common in rocks obtained from drill cores that reached 1000 metres depth.’’ The authors should be more careful in phrasing the implications of their work.

Since Maoniuping is an unusual case of carbonatite system, as the authors themselves suggest, the significance of this

study perhaps needs to be boasted less, at least the authors should not generalise the significance to the entire carbonatite system, but more towards presenting a fuller picture of carbonatite system that contains odd examples (line 99-103).

(2) The argument against a hydrothermal genesis of the REE mineralisation at Maoniuping is built on a shaky ground. The authors mis-interpret the references on previous inclusion work (line 73) and use the misinterpretation to doubt the validity of previous study. See comment on point (4)-misinterpretation of references.

Fluid inclusions in many hydrothermal fluids do not always contain ore minerals (Line 73-74). For example, gold rarely occurs as daughter minerals in the quartz fluid inclusions in the quartz-vein type. The lack of REE daughter minerals in inclusions is not sufficient to argue against a hydrothermal origin. If, according to the authors, the fluid inclusions are actually low T brine-melt inclusions (line 95), the fact that those inclusions are absent in ore minerals still argues against the validity of a brine-melt model for REE mineralisation.

Then the authors concern about a mixture of brine-melt and fluid phases in the sample inclusions, which can lead to erroneous interpretation (line 97). What would you expect to observe if the brine-melt and fluid phases are trapped in the same inclusions? Which mineral would be an ideal host mineral? And what phases would you expect in such an inclusion; H_2O plus abundant halite and REE minerals? And, has any Raman work suggested a hint of this speculation?

(3) The authors try to introduce a theory to explain the formation of Maoniuping, which needs substantial revision and clarification.
Reaction freezing and isothermal reactions
What does ''freezing'' exactly mean? The authors will first need to introduce the term before using it. This seems to be a discussion point, not something for the result section (line 216). Reaction freezing is brought up again in the discussion (line 421) but still not clearly explained. Do the authors mean the prevention of forming Na-K soluble minerals? According to their antiskarn theory, although formation of Na-K soluble minerals is restrained, reactions to form bastnäsite, baryte, and fluorite are actively going on (and simultaneously!), so reactions in the system do not seem to freeze.

The authors also advocate isothermal reactions, a theory with many flaws. The reactions presented in the Discussion section occur over decreasing temperature as the authors noted themselves (line 260-261), so not isothermal. The zonation of the pyroxenes is consistent with thermodynamic modelling of a cooling system (see line 259), so not isothermal. Removal of Na and K from the carbonatite melt is supposed to reduce the temperature (line 331-332), so not isothermal. Other evidence arguing against an isothermal or magma freezing process is presented in the comment to figure 6.

Brine-melts and antiskarn-albitisation
The formation of brine-melts should be briefly discussed in the Discussion section to give clarity. They could have formed due to the fractionation of calcite at depth (as the authors suggest). Another possibility is that the brine-melts only formed near the contact of the carbonatite with the syenite. Due to the metasomatism (albitisation + antiskarn formation), Ca and Mg are lost to the pyroxenes (Ca-Mg-rich cores). The alkalis are removed from the melt and incorporated into albite + pyroxene and amphibole (alkali-rich rims). What is left is a ''brine-melt'' that eventually crystallised as ore veins.

The process of antiskarn formation also needs more elaboration and revision at places.
Line 207-208: A crystallisation temperature for the Maoniuping crystals is not given in the text. Provide a temperature range.
Line 219-220: The observations only focus on the ore veins associated with a px-amp zone. Therefore, a magmatic formation can only be suggested for these zones/veins.
Line 223-225: It seems obvious that these low-temperature brine melts exist at shallower depths, near the edge of the intrusion. The ''barren'' carbonatites could just be the result of high-temperature crystallisation of the less evolved melt. They are barren because the REE are distributed over a large volume.
Line 245: The bleached zones also lack K-feldspar. Did the potassium end up in the phlogopite? And maybe some minor K partitioned into the albite/anorthoclase?
Line 279-281: This indicates that the formation of antiskarns is most likely a local phenomenon at shallower depths, instead of being responsible for the crystallisation of the entire carbonatite intrusion (as stated in line 415-416).
Line 292-296: The consistent presence of antiskarn-albitisation pair is also not a convincing theory. According to the authors, the pyroxene antiskarn is always observed together with an albitised zone. Some of the figures in this manuscript and references (e.g. 81) do not actually support this. The authors will need to present a realistic estimate of the prevalence of the antiskarn and albitised zones, respectively, and their spatial relationship at a deposit scale, if they wish to continue using this theory to explain the formation of entire Maoniuping.
Line 320: Do you suggest that the antiskarn-forming reaction occurred at 850°C or around/below? I don't think you have reliable temperature information for the anti-skarn reaction. The melt inclusion T in reference 29 seems to be wrong; while the feldspar pairs are actually hosted in the granite, instead of carbonatite or ore vein.
Line 332-336: The authors suggest the existence of a triggering point, however, the process is more likely to be gradual. The removal of alkalis leads to the gradual crystallisation of the carbonatite, with alkalis being depleted from the outer rim (forming antiskarns) inward. As a result, the carbonatite intrusion would solidify progressively, starting from the outer boundaries and moving toward the core. This process doesn't start when alkalis are ''wholly sequestered'', it starts the moment the first alkalis are removed.
The point made in the manuscript is clear:
-The alkalis (Na, K) are removed from the melt as they react with the silicate host rock.
- Consequently, the melt becomes richer in the (Ca, Mg)CO_3 component (more towards the right of the diagram in Fig. 6a).
- A carbonatite melt enriched in the (Ca, Mg)CO_3 component (depleted in alkalis) has a higher liquidus, thus starts to crystallise.

As this process occurs when the hot melt interacts with the cold(er) host rock, heat would be transferred to the country rock (i.e. cooling). The process would only be isothermal if the heat released by crystallisation equals the heat loss to the country rock. Any evidence for that?
Line 330-332: Another quantitative estimate to be made is the volume of ferromagnesian minerals formed from the amount of alkali carbonate removal. Is the scale close to the reality in Maoniuping?
Line 400-402: The authors suggest that the introduced silica glues all components in the system. This contradicts to what the authors proposed in Lines 253-255, where mass exchanges including Na, K, Al, Fe have occurred between the syenite and brine-melt.
Line 413-415: How does Fig. 4a (composition of Amp) support the ''solidification of the residual melt in an actively alkali-depleting melt system''? Instead, Fig. 4a only supports the first part of this sentence; the removal of alkalis to the refractory silicates (e.g. Amp + Px)

Antiskarn and REE mineralisation
Line 341-344: The authors attribute pegmatitic crystal growth (line 343) to the gas-dominated volatile phase (hydrothermal fluids). The REE are thought to remain in the brine-melt (line 340). However, the ore veins, suggested to have crystallised from the REE-enriched brine-melt, contain pegmatitic REE-bearing crystals such as bastnäsite (line 154 and line 393-394). Did the pegmatitic bastnäsite form from the brine-melt or from the ''low-density gas-dominated volatile phase''? The composition (REE-bearing) implies the former, while the crystal habit (pegmatitic) and texture (breccia) suggests the latter. This is contradicting.
Line 349: The authors suggest that the residual hydrothermal fluids are captured as fluid inclusions in minerals. That sounds unrealistic. In what minerals? If the minerals have already crystallised during the (early) magmatic phase, they can't capture late-stage hydrothermal fluids. Moreover, if part of the hydrothermal fluids remained uncaptured, what would occur to those escaped fluids? Fluid-mineral reactions? Any dissolution and replacement textures? Or formation of hydrothermal minerals?
Line 353-354: Please elaborate on this process and also discuss the role of brine-melt in this scenario.
Line 349-350: If all mineralisation formed magmatically, how did the minerals incorporate fluid inclusions? Fluid/melt inclusions are entrained during crystal growth? Furthermore, this contradicts with the authors' previous statement: Line 94 - 95: ''Consequently, previous studies have likely misclassified magmatic low-temperature brine-melt inclusions as "fluid inclusions.''
If the fluid inclusions are indeed brine-melt inclusions, this would suggest that they are enriched in REE? As stated in line 345: ''Crucially, ore-forming chemical components are primarily carried by the magmatic brine-melt, and not the dilute gas (i.e., the hydrothermal fluid).''
Line 419: 'Maoniuping veins'- Maoniuping shows a variety of mineralisation styles, including thin and thick veins and stockwork. Be rigorous when describing the Maoniuping geology . Also, a lack of surface exposure of ''surrounding distal fenites'' does not necessarily mean that these are not present at deeper parts of the system.
Line 420: Entirely? The authors will need to present a quantitative estimate of the occurrence of antiskarn and their spatial link to the REE mineralisation before drawing this conclusion.
Line 425-427: Reaction freezing near the edges is also the case for the pyroxene zones at Maoniuping. The pyroxene zones (''antiskarns'') are only found at the surface, in close association with albitised (fenitised) syenite. Is there evidence to suggest the ''metasomatic freezing'' occurred far away from the contact zone with the syenite?
Line 438-449: Is the silica-rich host rock also albitised at these other localities? Or is there any other evidence that suggest the introduction of silica into the carbonatite? As stated in line 292, the formation of antiskarns is apparently coupled to albitisation.
Line 453: Maoniuping may not represent an end member of complete interaction. The antiskarn formation at Maoniuping is only observed at the surface. Furthermore, the reactions that drive antiskarn formation are highly pressure-dependent (line 277). This seems to be continuum from deep emplacement (no antiskarns) to shallower emplacement depths (antiskarns). A single carbonatite intrusion could exhibit antiskarns at shallower depths, while the deep-seated core was not affected by the process. Depending on the present-day erosional level, you would either find antiskarns (limited erosion) or not at all (highly eroded; only deeper parts are exposed).
Line 455: The reaction degree of the brine-melt with silica rich rocks may also play a key role in determining how much mineralisation can be produced, instead of only a volume thing.
Line 461-462: What causes the carbonatite melt to intrude as thin veins? The proposed continuum seems to be one of erosional level. As shown in Vasyukova & Williams-Jones (2022). Deeper => massive carbonatite intrusions (ring complexes) Shallower => thin veins that originate from a deeper carbonatite body
Line 464-467: Rephrase this sentence to better summarize the conclusion. Ideally with additional information of geological indications, such as the host rock, possibly structural control, brine-melt and wall rock interaction degrees, and etc

(4) The authors need to check their references more carefully and critically. At many places the authors mis-interpret referred articles for their own favour and tend to misguide readers.
Line 68: Reference 30 actually also shows melt and melt-fluid inclusions, in addition to fluid inclusions. The authors of reference 30 suggest the melt and melt-fluid inclusions are magmatic, whereas the fluid inclusions are hydrothermal products. The authors of the manuscript simply ascribe the fluid inclusion to the product of synmagmatic fluids without reasoning.
Line 73: Simply wrong reference of original work. The fluid inclusion study in reference 30 actually was based on data from measuring fluorite, quartz, bastnaesite, calcite, barite.
Line 91: Couldn't find the key information of '2-2.4 kbar' in the reference paper.
Line 140: Please add which figures in each of the reference papers show the well-known pyroxene coats. Fig. 2b in reference 35 actually shows the absence of a pyroxene reaction zone between syenite and carbonatite. The feature is also not clearly noted in reference 34. In reference 24, the pyroxene coats are not obvious; fig. 3d indeed shows some kind of zonation, which is distinct from the pyroxene coats feature in this work and has distinct mineral assemblages (pyrite and K-

feldspar).
Line 276: In reference 24, the perthite occur in the alkaline granite, instead of the carbonatite or the reaction zone. Misuse of references.
Line 279: In reference 29, the methodology section states that ‘The measurable temperature range was −196 to +600°C’, but in their result section, temperature higher than 600 oC was reported.
Line 326-329: In reference 43, Muluozhai is quite different from this studied deposit in terms of mineral assemblages and wallrock type. The authors will need to give more detailed information of the melt inclusions in Muluozhai, including the host mineral, from what unit (from pyroxene-dominated zones or in ore veins?), the composition and homogenisation T.
Line 293: In reference 81, the figures actually show either the presence of albitisation or the antiskarn, instead of the presence of both always.
Line 339: In reference 24, the measured calcite is actually from the calcite carbonatite. The authors of this manuscript use the data of this calcite from the carbonatite magma, which is an earlier phase than the brine-melt, to discuss about the brine-melt with syenite, and the antiskarn formation and the degassing process. The authors will first need to establish a reasonable link of their own work to previous work before using their results for discussion.
Line 444: In all references 102-104, the authors suggest the REE mineralization is minor in the Wu dyke, and REE minerals occur in aggregates, suggesting they were primary minerals crystallizing directly from carbonatite magma. In reference 104, Fig.3d is are used for showing the texture and crystal size of bastnäsite, parisite and monazite, with associated baryte and fluorite, which is very different from the Maoniuping. If, according to the theory of this manuscirpt, those mineralisation is also formed from brine-melt, we would expect albitisation, breccia type mineralisation (degassing), mineral assemblage zoning, coarse and well crystalised REE minerals and abundant bastnäsite, baryte, and fluorite. Instead, the REE mineralisation in Wu dyke has abundant bastnäsite in aggregates and in association with quartz, calcite. It is a pre-mature conclusion that the authors try to conclude by comparing the Wu dyke and Maoniuping.

(5) The geology, petrology and mineralogy work of Maoniuping still requires a significant improvement.
The temporal and spatial relationships of the carbonatite magma, brine-melt and hydrothermal fluids at Maoniuping need clarification first and foremost. It is often not clear what rock unit the authors discuss about.
Line 131: Carbonatite only occurs at a very small outcrop in the Guangdoushan open pit. The authors need to note the location of samples in the figures of this study. Throughout the context, the authors should not generalise their work to the entire Maoniuping, because it contains two open pits which have varied geology, and the mineralisation style varies at depth.
Line 131-132: What is the spatial relationship of the carbonatite and ore vein? And what are their mineralogical differences/similarities?
Line 134: The Maoniuping seems to have > 20 ore veins in contact with syenite (and granite). Figure 2a-d can hardly explain the ubiquities of pyroxene of aegirine-augite and aegirine. Can you provide an estimate of the ubiquities of the occurrence? Furthermore, Fig. 2a actually shows the pyroxene develops at the carbonatite-syenite contact, instead of the vein-syenite contact. For the real ore vein-syenite contact, the pyroxene zone is either not obvious or missing in Figs. 2b and 2c.
Line 137-139: Detailed field photos or thin section micrographs need to be provided to show the growth direction.
Line 158: The authors also make this ‘’ore vein-carbonatite’’ distinction in their figures and text. They should clarify what they define as ‘’ore veins’’ before demonstrating the textural relationships in the figures.
Line 163-163: Please add thin section petrography work as supplement to support this statement. Hardly see mineral textures from Fig.2.
Line 166-168: Crystal shapes cannot be determined from these photos.

(6) Figures are poorly illustrated or organised. Suggestions for improvement are listed below.
Figure 1: Define where and which open pits these outcrops are located.
Figure 1b: Quantify how common this is? And do the ore veins also contain cpx? Or only around the edges? What are the spatial relationships of the pyroxene band with the ore veins? Are the ore veins always associated with a pyroxene band?
Figure 2: This figure is used as evidence that pyroxene grows outwards (within the carbonatite conduit), a feature which is not well visible from this figure. The authors need to provide more detailed images. Add a table of the mineralogical composition and proportions of the different units.
Figure 2: In Fig. 2a, pyroxene-amphibole band forms, but there seems to be no REE mineralisation in the calcite carbonatite. Whereas in Fig. 2d there is REE mineralisation in the so called ‘ore vein’ in association with pyroxene band. What causes this difference?
Figure 3: Provide a thin section to show the transition from antiskarn to Ca-rich carbonatite. A gradual change would be expected if the antiskarn formed from the carbonatite melt. What does white represent in figure 3b and 3c? There is a yellow rectangle on the right bottom in figure 3c- delete it. Rotate either fig. 3a or 3b or 3c or make the orientation aligned.
Figure 4: Please add a scan of the thin section either in figure 4 or as a supplement, which shows the co-occurrence relationship of REE minerals and those silicate minerals being measured. Please consider adding electron probe data for a line across the pyroxene crystal in the elemental maps, to have more quantitative assurance of the Na/K/Fe/Mg variation (line 184). Need to re-organise the order of plates. Fig. 4g goes before fig. 4c. Also need to state where this sample is from, i.e. ore vein. Be consistent with naming cpx or aeg-aug in the plates. What colour scale for the WDS elemental maps? Pinkish = higher concentration?
Figure 5: Please list all the elements on the X-axis. The REE element pattern is more like a humped sinusoidal pattern, instead of sinusoidal. Also note that in reference 54, the REE element pattern in fig.6 is primitive mantle normalised, while your data is Chondrite normalised.
Fig. 5a: Where are these samples from? What mineral assemblage? Add literature data on Cpx-Amp REE patterns from carbonatite and non-carbonatite (i.e. silicate) magmas.
Fig. 5b: Where do these fluorite-calcite pairs originate from? What rock, lithological unit, location? Please consider providing a thin section to show the mineral assemblage and the textural relationship to demonstrate the co-genetic relationship

between fluorite and calcite. Hard to judge what this data represents. The authors also need to explain more explicitly in the context what element activities might have caused the order of difference (line 209) and how the experimental set-up compares to Maoniuping.
Figure 6: This figure aims to illustrate a new crystallisation process. However, the metasomatic freezing mechanism involves the removal of alkalis by the introduction of new components into the system (predominantly Si). As the composition of the melt is externally changed, this defies the point of using a eutectic (homogeneous) system to explain the process? This is not a simple crystallisation process; it involves changing the melt composition through metasomatism. I don't think you can visualise this on such a binary phase diagram. The authors can consider using a different figure, maybe experimental figures would be better, to show how the temperature remains stable while antiskarns are forming.

Other minor comments are listed below and added in the annotated manuscript. A couple of places are missing key references and flagged out in the annotated manuscript.

Title: The title doesn't reflect the discussion points of this manuscript. Either magma solidification or reaction freezing is sufficiently explained in the context.

Abstract: Add how REE mineralisation is related to the antiskarn formation and wall rock contamination.

Introduction: Need more introduction on magmatic metasomatism, antiskarn and the genetic relationship of carbonatite magma, brine-melt and hydrothermal fluids in carbonatite REE system in general.
Line 11: The authors are mixing things up. Magmas do not solidify as a result of degassing. The solidification process is only temperature driven. The volatile degassing is an effect of exsolution by decompression.
Line 12 - 13: Not accurate. Magmatic metasomatism involves a silicate melt phase reacting with host rock. It is not only about H2O: see page 282-283: https://link.springer.com/article/10.1007/BF00307287
H2O is emphasised here for its role in forming metasomatic fluids, but in the later discussion section, H2O is barely discussed but instead SO2 and CO2 were discussed.
Line 26-27: I don't think this manuscript has provided sufficient evidence to conclude the relative importance of wall rock contamination and T decrease/volatile degassing. Modelling may provide some insight.
Line 34-35: This is not a description of process. This is a description of fact (if that is true).
Line 41: What about FeCO3? Since ankerite is mentioned in Line 44.
Line 66: It is an odd turning. The end of last paragraph presents a controversy on whether the ore-host rock is a carbonatite or hydrothermal rock. The following paragraph then starts with 'as with most carbonatite..' Abundant fluid inclusions can also exist in hydrothermal rocks. Don't misguide readers.

Results:
Line 179: 'Commonly' is overstated. Only one amphibole crystal was measured by EPMA spot, and shows difference in chemical composition. In the WDS element maps, the two Amp crystals show moderate enrichment of Ca at the rim, but for other elements, the zoning is not obvious.
Line 184: Please consider adding electron probe data for a line across the pyroxene crystal in the elemental maps, to have more quantitative assurance.

Discussion:
Please provide decent petrographic evidence to show the minerals growth pattern and paragenetic sequence, especially the growth direction of pyroxene. Contradicting points were made in terms of the paragenesis. The authors suggest 'amphibole and phlogopite most likely formed at the same time (line 247)', which seems to contradict with line 163: ''Within veins, amphibole formed first..''
The authors will need to present a mass balance estimate to support the theory based on reaction 2 (line 262-265). The pyroxene reaction zone doesn't seem to be prevalent geologically and cannot consume a huge amount of Fe to explain the lack of magnetite.
The authors will also need to provide a larger area of micro-XRF scan than figure 3 to demonstrate a transitional zone from the pyroxene reaction zone to calcite carbonate (or calcite abundant area in the ore vein). The petrographic evidence will be essential to support the reaction series and conclusions in line 272-274.
What is the discussion on feldspar all about? To suggest their formation in a hot environment? And what does it have to do with the antiskarn formation? If the authors want to use the feldspar discussion to support their point, they need to provide their own petrographic evidence of co-genesis of the two distinct feldspar and their co-genesis with the REE minerals.

Reviewer #3

(Remarks to the Author)
I co-reviewed this manuscript with one of the reviewers who provided the listed reports. This is part of the Nature Communications initiative to facilitate training in peer review and to provide appropriate recognition for Early Career Researchers who co-review manuscripts.

Version 2:

Reviewer comments:

Reviewer #2

(Remarks to the Author)
Review report

The revised manuscript is clearer and more readable than its previous version. While the majority of our concerns have been addressed, some issues still remain.

Major comment:
Figure 6a is not in its appropriate use. Consider deleting it or use another phase diagram. The binary eutectic phase diagram is normally used for an iso-chemical system. It is okay to use the diagram to describe the change of carbonatite melt from A to B, but not from B to C. During this stage, according to the authors, alkalis are removed from the carbonatite melt or brine-melt through reactions with the host rock, a process that is accompanied by the incorporation of Si and Al (± K) into the melt. This is no longer an iso-chemical system, nor a pure magmatic system, and the temperature doesn't stay the same.

From B to C, the diagram gives the illusion that the (Na,K)2CO3 component of the initial melt is removed, hence enriching the fluid in the (Ca,Mg)CO3 component. The only way to achieve that in such a binary eutectic diagram is through fractional crystallisation of pure Na-K carbonates. But that is not what is happening. In fact, only the alkalis are removed, and not in the form of (Na,K)2CO3 as the diagram suggests. Instead, the alkalis are sequestered in silicates. The externally added silica and aluminium were not part of the starting composition, and do not fit anywhere in this two-component carbonatite melt system. In other words, from A to B occurs within a closed carbonatite melt system (two-component), whereas the transition from B to C takes place in an open and reactive (multi-component) magmatic-metasomatic system. Hence, this not a suitable figure to visualize this process.

Minor comments:
Line 39: What do you mean by 'proper' hydrothermal fluids?
Line 123: ''a purely magmatic model''. The model that the authors propose lacks hydrous REE-enriched fluids, but is definitely not purely magmatic. It involves solid-melt element exchanges, i.e. contaminating the melt through metasomatism.
Line 197: However, the discussion on LREE partitioning in lines 267-270 seems to assume calcite-fluorite pair crystalises earlier than the fluorite-bastnasite pair.
Line 279-281: Awkward sentence- rephrase.
Line 290: K-feldspar is also consumed.
Line 338: In addition to silica, aluminium (and possibly K) is also involved in the exchange.
Line 371: Give the quartz context. Quartz is not mentioned to the petrography of either anti-skarn or the vein interiors. Are the studied quartz with melt inclusions associated with late hydrothermal phases?
Line 385: ''a non-isothermal environment'' – but this is not what B to C in figure 6b shows.
Line 494: Move the sentence ''Elsewhere, bastnäsite … in burbankite'' to before line 492 (''At Maoniuping..''). First describe how bastnäsite typically forms, then discuss why this can't be the case for Maoniuping.
Line 533: It is not a frozen but a contaminated brine-melt.
Line 565: It is a local phenomenon at Maoniuping.

Laurens Tromp & Lingli Zhou

Reviewer #3

(Remarks to the Author)
I co-reviewed this manuscript with one of the reviewers who provided the listed reports. This is part of the Nature Communications initiative to facilitate training in peer review and to provide appropriate recognition for Early Career Researchers who co-review manuscripts.

Open Access This Peer Review File is licensed under a Creative Commons Attribution 4.0 International License, which permits use, sharing, adaptation, distribution and reproduction in any medium or format, as long as you give appropriate credit to the original author(s) and the source, provide a link to the Creative Commons license, and indicate if changes were made.
In cases where reviewers are anonymous, credit should be given to 'Anonymous Referee' and the source.
The images or other third party material in this Peer Review File are included in the article’s Creative Commons license, unless indicated otherwise in a credit line to the material. If material is not included in the article’s Creative Commons license and your intended use is not permitted by statutory regulation or exceeds the permitted use, you will need to obtain permission directly from the copyright holder.
To view a copy of this license, visit https://creativecommons.org/licenses/by/4.0/

Response to reviewers

Reviewer comments in black and serif font.

Our response in orange and sans serif font.

Reviewer #1 (Remarks to the Author):

The submitted manuscript represents a strongly revised version of a manuscript, which I previously reviewed. Most of the concerns I raised back then (March 2022) are well addressed now and apart from few minor things I do not have any major objectives anymore. All of my comments are given in the attached manuscript file and I hope that they are of some use. I recommend publication of this manuscript after minor revisions.

Thank you. Answers to issues raised in the annotated version of the manuscript for Reviewer 1 and reviewer 3 are directly added to Word files.

Reviewer #2 (Remarks to the Author):

Review report on 'Magma solidification via reaction freezing at the Maoniuping carbonatite'

Please note all line numbers are based on the annotated Word document opened on Mac. Minor changes may occur in a Window system.

(1) Significance and focus of this study

The goal of this study is to demonstrate that the pyroxene-amphibole zones (antiskarns) are linked to the vein-type REE mineralisation style at Maoniuping.

Not exactly. We use this observation to demonstrate a novel magmatic process. This point will be repeated several times here, and also explained in the cover letter.

Instead of a hydrothermal origin, the authors propose that the mineralisation formed magmatically from low-temperature brine-melts that metasomatized the country rock. While the formation of antiskarns via metasomatic exchanges between a brine-melt and the country rock seems reasonable, the (potential) genetic link between the antiskarns and the REE mineralisation has not been thoroughly investigated. The simultaneous antiskarn formation and ore-vein crystallisation should first be confirmed, before concluding that the two are inherently related. Additional geological/petrographic/geochemical evidence is required to support this hypothesis.

Additional evidence provided, see below and in the revised manuscript for specific details.

In addition, the authors suggest that the REE mineralisation formed by isothermal ''metasomatic freezing''. This is a novel petrological process that, according to the authors, results in the isothermal solidification of a melt. Although the general mechanism (removal of alkali fluxes from the melt) is plausible, the thermodynamic evidence has not been provided. Primary crystallisation temperatures for the antiskarns and the REE ore-veins should also be determined to support this claim. Furthermore, the authors appear to contradict themselves on the isothermal nature of this process.

This is now clarified in the manuscript. We removed the reference to "freezing" as this implies non-isothermal process, and can be interpreted differently by other people. Although "freezing" sounds good, we now just talk about "solidification" to remove any ambiguities. It's important to note that in reality, the process is unlikely to be isothermal as this is an evolving magmatic system with crystallisation-driven latent heat release, non-equilibrium reactions between different thermal bodies, etc. The point is that the

process does not require any temperature change (or pressure, for that matter) in contrast to most commonly accepted magmatic models. In order words, in the *P*–*T*–*X* thermodynamic space, the previously known crystallisation models are driven by changes in *P* and *T*, but here we show a model driven by changes in *X*. This is the big story here, and the manuscript has been revised throughout to clarify this.

All in all, the authors address some important and relevant points that definitely deserve attention. However, the authors draw some major conclusions from rather limited lines of evidence, and particularly lack reliable geology information derived from their own study. For example, the ''entire formation'' of Maoniuping has been suggested to be the result of ''antiskarn forming reactions and resulting reaction freezing'', while only a few outcrops have been studied. The authors also state themselves that ''evidence for these reactions is much less common in rocks obtained from drill cores that reached 1000 metres depth.'' The authors should be more careful in phrasing the implications of their work.

That is a fair comment – we should not talk about "entire formation of Maoniuping" as a blanket statement. We now focus this to the shallow mineralised REE-rich zones. However, we emphasise that REE mineralisation without any antiskarns is extremely uncommon at Maoniuping.

Since Maoniuping is an unusual case of carbonatite system, as the authors themselves suggest, the significance of this study perhaps needs to be boasted less, at least the authors should not generalise the significance to the entire carbonatite system, but more towards presenting a fuller picture of carbonatite system that contains odd examples (line 99-103).

We disagree. Maoniuping is an endmember case, and is therefore informative. From our experience, almost every other carbonatite in the world has elements in it which are like Maoniuping. Several salient examples are given in the paper, but it is not our role or in out scope to point out every single petrographic observation in carbonatites that should be reinterpreted based on our model. We will leave that to future workers to do. That said, we again emphasise that the point of this manuscript is not to explain the formation of Maoniuping. It is to use Maoniuping as an example to demonstrate a novel magmatic crystallisation process. Claims that we do not fully explain Maoniuping, and there are places (deeper, elsewhere, some veins in a different mining pit) that do not fully conform to our model are missing the point of the manuscript. Of course that not every single rock in Maoniuping will be explained by our model. However, our model leads to a fundamental change in the understanding of many rocks at Maoniuping, and by extension, elsewhere in the world.

(2) The argument against a hydrothermal genesis of the REE mineralisation at Maoniuping is built on a shaky ground.

The authors mis-interpret the references on previous inclusion work (line 73) and use the misinterpretation to doubt the validity of previous study. See comment on point (4)-misinterpretation of references.

No, there is no misinterpretation. We have discussed this point repeatedly in our previous submission of this manuscript, reviewed by one of the authors of that very same paper. We include our response here, to the right (reviewer in orange, our reply in dark blue). We stand by our statement that the original Xie 2015 "finding" of a REE-rich fluid is incorrect. Maoniuping is one of the best studied carbonatite localities in the world, and since that paper not a single report of fluid inclusions has managed to replicate the finding of a REE-rich *fluid* inclusion (as distinct from brine-melt). If the reviewer or other believe that REE mobility in Maoniuping was driven by hydrothermal fluids, we invite them to publish a paper demonstrating that, instead of relying on a single erroneous and nonreproducible decade-old report. We would also add that REE-solubility in fluids have been studied experimentally quite extensively in the past decade, and no study* had found any notable REE-solubility in the hydrothermal fluids like the ones in Maoniuping (*except from studies based on the erroneous Xie 2015 report). As this point is already made very clear in our manuscript, no revision is required here. We did, however, add some general statements on the overall (un)suitability of fluid inclusions studies to resolve these issues.

(28)Line 164-166: "However, all Maoniuping fluid inclusion studies over the past decade have failed to find any —ore, or notable REE contents, inside the inclusions[4]"

High REE content has been confirmed in fluid, melt fluid inclusion in Maoniuping (Xie et al., 2015). Xie et al., 2015 have reported high REE content in the melt-fluid inclusion hosted in fluorite using LA-ICP-MS.

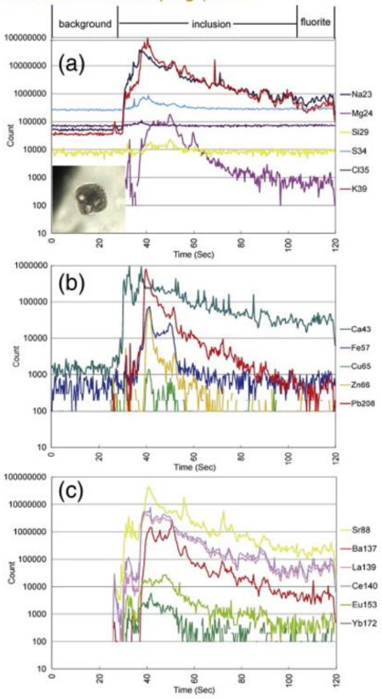

This is figure 18 from Xie et al 2015. Note at the top how the authors marked the whole time period from 30 to 105 sec as the inclusion. However, when looking at the actual signal and looking at elements which are both fluid mobile and incompatible in fluorite (ie, things which would otherwise be expected in a fluid inclusion) you can see that S, Cl, Pb, Zn, Cu all rapidly increase at 40 sec and drop off rapidly by 60 sec. To us, this means that most of the signal marked as "inclusion" by Xie et al 2015 isn't actually an inclusion, and is mostly the background fluorite. Note that the signal and slope of the REE La and Ce is essentially identical to that of Sr and Ca, which are the matrix elements of the host fluorite, and are consistent throughout the ablation signal. There is no spike in the REE signal between 40 and 60 seconds, when the inclusion was ablated. Therefore, we reckon that the REE signal is of the host fluorite and not of the inclusion, and consequently, there is no evidence for REE in the *fluid* inclusion.

As for REE in *melt* inclusions, this is absolutely fine because we suggest a magmatic process where the REE are precipitated from the melt.

Chen et al. (2021) evaluated the REE content in the primary carbonatite fluid.

No, they didn't. Chen et al is an experimental study of potassium sulfate, not REE. The REE evaluation is based on a comparison with the data of Xie et al 2015, which we consider to be flawed (see above).

Fluid inclusions in many hydrothermal fluids do not always contain ore minerals (Line 73-74). For example, gold rarely occurs as daughter minerals in the quartz fluid inclusions in the quartz-vein type. The lack of REE daughter minerals in inclusions is not sufficient to argue against a hydrothermal origin. If, according to the authors, the fluid inclusions are actually low T brine-melt inclusions (line 95), the fact that those inclusions are absent in ore minerals still argues against the validity of a brine-melt model for REE mineralisation.

Using gold is not the best example, because the solubility of gold is so small that even when present in a fluid inclusion, it is not in sufficient abundance to form observable daughter minerals. The claim of absence of brine-melt inclusions as evidence against is again not ideal, because we know from thermodynamics and experimental studies which fluids and which brine-melts can dissolve REE in carbonatite systems. REE-deposits don't magically appear – the REE has to come from somewhere. Even when inclusions are not present, residual minerals and experimentally-founded thermodynamic evidence can form a basis for an interpretation (as is thoroughly discussed in the second half of our

Discussion). We know it cannot be just a "hydrothermal fluid", and the only other model that exists as of 2025 is brine-melts. As above, this information is already in the manuscript, and no revision is made. If the reviewers (or someone else) wish to claim that hydrothermal fluids can mobilise REE, please present the thermodynamic data that it is possible in the chemical system buffered by the minerals present at Maoniuping.

Then the authors concern about a mixture of brine-melt and fluid phases in the sample inclusions, which can lead to erroneous interpretation (line 97). What would you expect to observe if the brine-melt and fluid phases are trapped in the same inclusions? Which mineral would be an ideal host mineral? And what phases would you expect in such an inclusion; H_2O plus abundant halite and REE minerals? And, has any Raman work suggested a hint of this speculation?

Good point – this has been clarified in the revised manuscript. Regarding an ideal host mineral – nothing really. The best host is quartz, but quartz does not form in the presence of a brine-melt. All other options are either REE-rich themselves (bastnasite, fluorite), obscuring the REE-rich nature of the inclusions, or poor containers of inclusions (calcite, baryte). Ideally, the silicates amphibole or pyroxene would work great, but they form at non-equilibrium from a contaminated (thus compromised brine-melt) and the composition would be biased. Note that brine-melts aren't necessarily halite-dominated. The most common anion would be carbonate, then followed by sulfate, chloride, etc. We are not sure how Raman would be useful in this case, as the reviewer didn't state exactly which question should be answered by Raman, and it is unclear what "this speculation" actually is. Nevertheless, this issue is now better explained in the manuscript.

(3) The authors try to introduce a theory to explain the formation of Maoniuping, which needs substantial revision and clarification.

Reaction freezing and isothermal reactions

What does ''freezing" exactly mean? The authors will first need to introduce the term before using it. This seems to be a discussion point, not something for the result section (line 216). Reaction freezing is brought up again in the discussion (line 421) but still not clearly explained. Do the authors mean the prevention of forming Na-K soluble minerals? According to their antiskarn theory, although formation of Na-K soluble minerals is restrained, reactions to form bastnäsite, baryte, and fluorite are actively going on (and simultaneously!), so reactions in the system do not seem to freeze.

As above, mentions of "freezing" are removed to avoid ambiguity. The reviewers have correctly understood our claim, so no revision is made according to the second half of this comment.

The authors also advocate isothermal reactions, a theory with many flaws. The reactions presented in the Discussion section occur over decreasing temperature as the authors noted themselves (line 260-261), so not isothermal. The zonation of the pyroxenes is consistent with thermodynamic modelling of a cooling system (see line 259), so not isothermal. Removal of Na and K from the carbonatite melt is supposed to reduce the temperature (line 331-332), so not isothermal. Other evidence arguing against an isothermal or magma freezing process is presented in the comment to figure 6.

We added a paragraph clarifying what do we mean by "isothermal". We note that the removal of Na and K from the melt to refractory minerals does not reduce temperature, it increases the melting point thus triggering a phase transition from liquid to solid. This is already clear enough in the manuscript, no revision done on this.

Brine-melts and antiskarn-albitisation

The formation of brine-melts should be briefly discussed in the Discussion section to give clarity. They could have formed due to the fractionation of calcite at depth (as the authors suggest). Another possibility is that the brine-melts only formed near the contact of the carbonatite with the syenite. Due to the metasomatism (albitisation + antiskarn formation), Ca and Mg are lost to the pyroxenes (Ca-Mg-rich cores). The alkalis are removed from the melt and incorporated into albite + pyroxene and amphibole (alkali-rich rims). What is left is a ''brine-melt'' that eventually crystallised as ore veins.

Yes, absolutely. This is very correct, and we did not want to get into too much depth initially, but you are absolutely right and it is useful to include this information in the paper. Manuscript is revised accordingly.

The process of antiskarn formation also needs more elaboration and revision at places.

Line 207-208: A crystallisation temperature for the Maoniuping crystals is not given in the text. Provide a temperature range.

Range of < 700 °C added to the text, based on feldspars (as opposed to feldspathoids) and literature melt inclusion studies.

Line 219-220: The observations only focus on the ore veins associated with a px-amp zone. Therefore, a magmatic formation can only be suggested for these zones/veins.

Yes, but this includes most ore veins as both pyroxene and amphiboles are ubiquitous the shallow parts of Maoniuping, particularly Dagudao. This is now clarified. Again, we note that REE without px zones are very rare at Maoniuping, so although this is not a unique crystallisation model, it is the most common one.

Line 223-225: It seems obvious that these low-temperature brine melts exist at shallower depths, near the edge of the intrusion. The ''barren'' carbonatites could just be the result of high-temperature crystallisation of the less evolved melt. They are barren because the REE are distributed over a large volume.

Yes, indeed, as is the case for almost all carbonatites in the world that contain mostly barren calcite carbonatites and mineralised portions are shallow and much more Mg-Fe rich. These shallow zones are exactly the parts that are Ba–REE–Sr-rich. This is already in the introduction and later on in the discussion, so no revision made.

Line 245: The bleached zones also lack K-feldspar. Did the potassium end up in the phlogopite? And maybe some minor K partitioned into the albite/anorthoclase?

Yes, this exact thing is explained a few sentences down.

Line 279-281: This indicates that the formation of antiskarns is most likely a local phenomenon at shallower depths, instead of being responsible for the crystallisation of the entire carbonatite intrusion (as stated in line 415-416).

Correct, the manuscript has been revised accordingly.

Line 292-296: The consistent presence of antiskarn-albitisation pair is also not a convincing theory. According to the authors, the pyroxene antiskarn is always observed together with an albitised zone. Some of the figures in this manuscript and references (e.g. 81) do not actually support this. The authors will need to present a realistic estimate of the prevalence of the antiskarn and albitised zones,

respectively, and their spatial relationship at a deposit scale, if they wish to continue using this theory to explain the formation of entire Maoniuping.

We no longer claim that this formed the entire Maoniuping complex. Nevertheless, we added relevant references demonstrating that the shallow part is indeed dominated by antiskarns. Demonstrating albitisation is much harder, because it is a feature that was mostly overlooked in previous studies, but here we show that it is indeed the case. We added annotations better pointing out bleached zones.

Line 320: Do you suggest that the antiskarn-forming reaction occurred at 850°C or around/below? I don't think you have reliable temperature information for the anti-skarn reaction. The melt inclusion T in reference 29 seems to be wrong; while the feldspar pairs are actually hosted in the granite, instead of carbonatite or ore vein.

Not 850 °C, the next sentence explains why this estimate is wrong. And then immediately after that we're talking about a range of 600 °C-ish based on thermodynamic constraints. Elsewhere in the paper the presence of perthites also constrains temperature to much lower than 850 °C, again supported by thermodynamics. We don't know why the reviewer states that ref 29 (Z&L 2019 OGR) is about a granite, not a carbonatite. It was co-authored by one of us (YL), and this is very clearly from the carbonatite. At no place in ref 29 does it say the feldspars are hosted in granite.

Line 332-336: The authors suggest the existence of a triggering point, however, the process is more likely to be gradual. The removal of alkalis leads to the gradual crystallisation of the carbonatite, with alkalis being depleted from the outer rim (forming antiskarns) inward. As a result, the carbonatite intrusion would solidify progressively, starting from the outer boundaries and moving toward the core. This process doesn't start when alkalis are ''wholly sequestered'', it starts the moment the first alkalis are removed.

Yes, this is correct. Manuscript revised to correct and clarify this.

The point made in the manuscript is clear:

-The alkalis (Na, K) are removed from the melt as they react with the silicate host rock.

- Consequently, the melt becomes richer in the (Ca, Mg)CO_3 component (more towards the right of the diagram in Fig. 6a).

- A carbonatite melt enriched in the (Ca, Mg)CO_3 component (depleted in alkalis) has a higher liquidus, thus starts to crystallise.

Yes, you are very much correct.

As this process occurs when the hot melt interacts with the cold(er) host rock, heat would be transferred to the country rock (i.e. cooling). The process would only be isothermal if the heat released by crystallisation equals the heat loss to the country rock. Any evidence for that?

As explained above, the isothermal process is clarified in the revised manuscript.

Line 330-332: Another quantitative estimate to be made is the volume of ferromagnesian minerals formed from the amount of alkali carbonate removal. Is the scale close to the reality in Maoniuping?

This is impossible to assess without knowing exactly how much brine-melt existed, what was its composition, and whether any has been lost. You're essentially asking us to derive

a fluid-melt/rock ratio when most parameters are unknown. This can be fudged until something comes out that makes sense, with low confidence. We prefer to not to do this, as this will be speculation disguised as quantification, and an astute reader will see through this easily.

Line 400-402: The authors suggest that the introduced silica glues all components in the system. This contradicts to what the authors proposed in Lines 253-255, where mass exchanges including Na, K, Al, Fe have occurred between the syenite and brine-melt.

How does this contradict? Most mass exchange is Si and Al going from the syenite to the carbonatite, where they "glue" Ca, Mg, K, and Na. Minor Na goes to albitise the syenite, where it is also glued by Si. Minor K is lost from the syenite, and is again glued by Si-Al-Mg to make phlogopite. This is exactly what we're saying, so we don't see the problem here. But you're right – some clarification is in order because Si does not glue *everything*. REE and Ba for example do not end up in silicates. The manuscript has been revised to address this.

Line 413-415: How does Fig. 4a (composition of Amp) support the ''solidification of the residual melt in an actively alkali-depleting melt system''? Instead, Fig. 4a only supports the first part of this sentence; the removal of alkalis to the refractory silicates (e.g. Amp + Px)

Correct, figure reference moved to a more correct and less confusing location.

Antiskarn and REE mineralisation

Line 341-344: The authors attribute pegmatitic crystal growth (line 343) to the gas-dominated volatile phase (hydrothermal fluids). The REE are thought to remain in the brine-melt (line 340). However, the ore veins, suggested to have crystallised from the REE-enriched brine-melt, contain pegmatitic REE-bearing crystals such as bastnäsite (line 154 and line 393-394). Did the pegmatitic bastnäsite form from the brine-melt or from the ''low-density gas-dominated volatile phase''? The composition (REE-bearing) implies the former, while the crystal habit (pegmatitic) and texture (breccia) suggests the latter. This is contradicting.

You're right, this is a bit confusing. What we meant is that minerals are crystallising from the brine-melt, but the presence of a coexisting gas permits the volume into which the crystals are forming. We admit this is somewhat speculative (and could even be wrong!), and does not contribute that much to the paper. This sentence is now removed.

Line 349: The authors suggest that the residual hydrothermal fluids are captured as fluid inclusions in minerals. That sounds unrealistic. In what minerals? If the minerals have already crystallised during the (early) magmatic phase, they can't capture late-stage hydrothermal fluids. Moreover, if part of the hydrothermal fluids remained uncaptured, what would occur to those escaped fluids? Fluid-mineral reactions? Any dissolution and replacement textures? Or formation of hydrothermal minerals?

Why unrealistic? Most hydrothermal fluid inclusions are hosted in quartz (which is a late hydrothermal mineral), or in the easily cleaved fluorite and bastnäsite, so these would be secondary inclusions disguised as primary since they're along cleavage planes masquerading as growth zones. This was discussed at length in fluid inclusion studies before (Xie 2019, Liu&Zheng 2019), and is appropriately referenced in the manuscript. The nature of fluid inclusions is now further clarified in the manuscript.

Regarding what would be the fate of the fluids and associated mineral assemblage, there would be not much happening. The last part of the manuscript is exactly about this – in most carbonatites the fluid alteration is the cause for the common bastnäsite–fluorite mineral association. At Maoniuping, this is already the assemblage that forms magmatically, already in equilibrium with any hydrothermal fluids, such that no alteration would occur. This is already rather clear in the manuscript, so no revisions done.

Line 353-354: Please elaborate on this process and also discuss the role of brine-melt in this scenario.

Assuming line numbers are correct, this part is talking about pH and acidic fluids. There isn't much more to say – the cited references talk about REE mobility in acidic fluids. Maoniuping is a calcite-buffered, alkaline and non-acidic system, hence REE are not mobile. The role of the brine-melt is that it's alkaline. Added a few words to clarify.

Line 349-350: If all mineralisation formed magmatically, how did the minerals incorporate fluid inclusions? Fluid/melt inclusions are entrained during crystal growth? Furthermore, this contradicts with the authors' previous statement: Line 94 - 95: ''Consequently, previous studies have likely misclassified magmatic low-temperature brine-melt inclusions as "fluid inclusions."

See answer above.

If the fluid inclusions are indeed brine-melt inclusions, this would suggest that they are enriched in REE? As stated in line 345: ''Crucially, ore-forming chemical components are primarily carried by the magmatic brine-melt, and not the dilute gas (i.e., the hydrothermal fluid)."

Potentially? Unfortunately, the only study that measured REE contents in fluid inclusions is the Xie study above, which we have issues with (see our comment earlier). The problem is that obvious melt inclusions are hosted in either bastnäsite or fluorite, both are REE-rich minerals. Their REE are more abundant that what would be expected in the melt inclusions, such that actually measuring REE in the inclusion is problematic. **<u>This is the crux of the magmatic–hydrothermal debate of Maoniuping</u>!** Usually, fluid or melt inclusions are very useful methods to reveal the presence of ore elements in fluids or melts. However, here melt inclusions are hosted in REE-rich minerals, and often mistaken as fluid inclusions. Fluid inclusions are not expected to contain any (according to our model) and indeed do not contain any. Therefore, fluid or melt inclusions are not a good tool to decipher the REE formation in Maoniuping and an alternative approach (i.e., ours) is required. This is now clarified in the manuscript.

Line 419: 'Maoniuping veins'- Maoniuping shows a variety of mineralisation styles, including thin and thick veins and stockwork. Be rigorous when describing the Maoniuping geology . Also, a lack of surface exposure of ''surrounding distal fenites" does not necessarily mean that these are not present at deeper parts of the system.

Clarified that this is not only on the surface, but also in deep drilling. Added some explanation on the various type of veins and relevant references. Regarding distal fenites, yes, absence of evidence is not evidence for absence, but at some point, if you're not finding something where you might expect it to be, then the sensible conclusion is that it's not there. Maoniuping is an active quarry with very active brownfield exploration around it. If distal fenite veins existed, they would have already been found.

Line 420: Entirely? The authors will need to present a quantitative estimate of the occurrence of antiskarn and their spatial link to the REE mineralisation before drawing this conclusion.

Yes, corrected.

Line 425-427: Reaction freezing near the edges is also the case for the pyroxene zones at Maoniuping. The pyroxene zones (''antiskarns'') are only found at the surface, in close association with albitised (fenitised) syenite. Is there evidence to suggest the ''metasomatic freezing'' occurred far away from the contact zone with the syenite?

At Maoniuping, there is no "far away". Most, if not all, dykes and veins are very thin, up to several metres. What this section describes is large circular carbonatite complexes where the intrusion is many tens to hundreds of metres across. An important point is that REE mineralisation is always immediately adjacent or mere centimetres away from antiskarn-related silicates. Now clarified in the manuscript.

Line 438-449: Is the silica-rich host rock also albitised at these other localities? Or is there any other evidence that suggest the introduction of silica into the carbonatite? As stated in line 292, the formation of antiskarns is apparently coupled to albitisation.

Excellent question. No one ever looks at the host rock, because people erroneously think that alteration ends at the "fenite", mistaking antiskarns as fenites. It is our hope that this study will encourage people to look further into the silica rocks and find this alteration. Note that at Maoniuping we see albitisation because it's a sodic system introducing into a very felsic quartz syenite, and it might be different in other systems with different chemical compositions. Similar host rocks exist all over the Mianning-Dechang belt so it is difficult to assess intrusions into different host rocks in our case. This is a very good point and now added to the manuscript. To your original question, we added some references mentioning albite in those rocks (albeit not proper albitisation like at Maoniuping).

Line 453: Maoniuping may not represent an end member of complete interaction. The antiskarn formation at Maoniuping is only observed at the surface. Furthermore, the reactions that drive antiskarn formation are highly pressure-dependent (line 277). This seems to be continuum from deep emplacement (no antiskarns) to shallower emplacement depths (antiskarns). A single carbonatite intrusion could exhibit antiskarns at shallower depths, while the deep-seated core was not affected by the process. Depending on the present-day erosional level, you would either find antiskarns (limited erosion) or not at all (highly eroded; only deeper parts are exposed).

Yes, correct. Note, however, that REE mineralisation is typically a shallow process, demonstrating an important role for host rock composition. Remember that REE-enrichment is typically only achieved after protracted fractionation, and almost always appears close to the surface in carbonatites worldwide. Deep REE mineralisation is rather rare, anywhere. This is now clarified and referenced in the manuscript.

Line 455: The reaction degree of the brine-melt with silica rich rocks may also play a key role in determining how much mineralisation can be produced, instead of only a volume thing.

Yes, this has been repeated several times in the manuscript.

Line 461-462: What causes the carbonatite melt to intrude as thin veins? The proposed continuum seems to be one of erosional level. As shown in Vasyukova & Williams-Jones (2022). Deeper => massive

carbonatite intrusions (ring complexes) Shallower => thin veins that originate from a deeper carbonatite body

Local stress fields? Pre-existing structures? Host rock type? Structural and geophysical assessment of intrusion dynamics is beyond the scope of this manuscript. Nonetheless, it is not a question of erosional level. Many other carbonatites that haven't reached the endmember conditions at Maoniuping *do have* REE mineralisation, but it looks different. This is already discussed in detail in the manuscript.

Line 464-467: Rephrase this sentence to better summarize the conclusion. Ideally with additional information of geological indications, such as the host rock, possibly structural control, brine-melt and wall rock interaction degrees, and etc

We choose to leave this sentence as is, but we have the major improvements we made in the previous paragraphs make this conclusion clearer, with a specific reference to host rocks and interaction degrees.

(4) The authors need to check their references more carefully and critically. At many places the authors mis-interpret referred articles for their own favour and tend to misguide readers.

Line 68: Reference 30 actually also shows melt and melt-fluid inclusions, in addition to fluid inclusions. The authors of reference 30 suggest the melt and melt-fluid inclusions are magmatic, whereas the fluid inclusions are hydrothermal products. The authors of the manuscript simply ascribe the fluid inclusion to the product of synmagmatic fluids without reasoning.

Yes, they have melt inclusions and naturally those would be magmatic. But their conclusion is that the mineralisation is hydrothermal, and this is what we're challenging here. We clarified what we mean by synmagmatic.

Line 73: Simply wrong reference of original work. The fluid inclusion study in reference 30 actually was based on data from measuring fluorite, quartz, bastnaesite, calcite, barite.

Yes, they worked on all those minerals. But the infamous REE-bearing fluid inclusions is hosted in fluorite. They did not find REE in inclusions hosted by other minerals.

Line 91: Couldn't find the key information of '2-2.4 kbar' in the reference paper.

Apologies – wrong paper cited. Changed to the correct one: abstract of Z&L 2019 clearly says: "pressures of 2.0–2.4 kbar" with rationale in the paper itself. Note that Zheng 2021 also infers 240 MPa (=2.4 kbar).

Line 140: Please add which figures in each of the reference papers show the well-known pyroxene coats. Fig. 2b in reference 35 actually shows the absence of a pyroxene reaction zone between syenite and carbonatite.

Yes, fig 2b does not show it, but fig 2a *does* show it. Note that the carbonatite in their 2b is not particularly mineralised. It's just mostly carbonate, so no antiskarn is expected there. As above, the point of this manuscript isn't to show that every single rock at Maoniuping has an antiskarn–albite pair with associated REE mineralisation. It is to show a process that occurs in many if not most rocks, but not all.

The feature is also not clearly noted in reference 34.

Guo & Liu 2019 shows exactly this, very clearly in their fig 4D.

In reference 24, the pyroxene coats are not obvious; fig. 3d indeed shows some kind of zonation, which is distinct from the pyroxene coats feature in this work and has distinct mineral assemblages (pyrite and K-feldspar).

First, this is a deeper sample from drilling so the extent of the reaction will be diminished. However, note the very clear syenite to feldspar to aegirine to carbonatite. This is very distinctive, and an excellent example of compositional control, where instead of albite+aegirine±phlogopite as in the shallow samples, there is now K-feldspar+phlogopite±aegirine. This is also added to the conclusion section.

Line 276: In reference 24, the perthite occur in the alkaline granite, instead of the carbonatite or the reaction zone. Misuse of references.

You are correct, ref 24 does not talk about perthite in the carbonatite. There are perthites in the rocks. We now added a figure to show this with a newly acquired TIMA map of perthite feldspar.

Line 279: In reference 29, the methodology section states that 'The measurable temperature range was −196 to +600°C', but in their result section, temperature higher than 600 oC was reported.

Their higher than 600 °C refers to the previous study by Xie 2009, which they cite appropriately. Their own data is just given as "> 600 °C" because that's the temperature range of their instruments. No issue here.

Line 326-329: In reference 43, Muluozhai is quite different from this studied deposit in terms of mineral assemblages and wallrock type. The authors will need to give more detailed information of the melt inclusions in Muluozhai, including the host mineral, from what unit (from pyroxene-dominated zones or in ore veins?), the composition and homogenisation T.

Observation of brine-melts in Muluozhai is very interesting, but going into the depth of explaining this will make the manuscript even longer. It is already long enough and reference-heavy, so we decided to remove this part completely as it does not actually contribute much to our manuscript.

Line 293: In reference 81, the figures actually show either the presence of albitisation or the antiskarn, instead of the presence of both always.

To be clear, there are no figures that show any rocks in ref 81 (Le Bas 2008). It is all based on the description in the text. This reference does not contribute much here, and therefore we removed it in order to lower the overall reference count of our manuscript.

Line 339: In reference 24, the measured calcite is actually from the calcite carbonatite. The authors of this manuscript use the data of this calcite from the carbonatite magma, which is an earlier phase than the brine-melt, to discuss about the brine-melt with syenite, and the antiskarn formation and the degassing process. The authors will first need to establish a reasonable link of their own work to previous work before using their results for discussion.

This is incorrect. Their calcite measured for isotopes is explicitly described as being from the reaction zones (see their bottom right of page 27, and data tables). The link is clear.

Line 444: In all references 102-104, the authors suggest the REE mineralization is minor in the Wu dyke, and REE minerals occur in aggregates, suggesting they were primary minerals crystallizing directly from carbonatite magma. In reference 104, Fig.3d is are used for showing the texture and crystal size of

bastnäsite, parisite and monazite, with associated baryte and fluorite, which is very different from the Maoniuping. If, according to the theory of this manuscirpt, those mineralisation is also formed from brine-melt, we would expect albitisation, breccia type mineralisation (degassing), mineral assemblage zoning, coarse and well crystalised REE minerals and abundant bastnäsite, baryte, and fluorite. Instead, the REE mineralisation in Wu dyke has abundant bastnäsite in aggregates and in association with quartz, calcite. It is a pre-mature conclusion that the authors try to conclude by comparing the Wu dyke and Maoniuping.

Crystal size and textures are irrelevant. Thermodynamics (which is what we're essentially proposing here) works regardless of crystal size. There is also no requirement for the presence of albite, or even zoning. Maoniuping shows it well, other places don't. This is why we use Maoniuping to demonstrate the process. The Wu dyke story is included here as an example of additional localities that can be reinterpreted using inferences from Maoniuping. This is already written several times in the manuscript, at different places.

(5) The geology, petrology and mineralogy work of Maoniuping still requires a significant improvement.

We will use this opportunity again to explain that the purpose of this paper is not to explain Maoniuping and provide a thorough description of its geology, petrology, and mineralogy. The purpose is to show a novel magmatic process, that is very obvious at Maoniuping (if you know where to look for and what to look at), show that this process also leads to magmatic REE mineralisation, and to show that it likely occurs in all carbonatites globally. As such, there is no need for us to describe the geology, petrology, and mineralogy of Maoniuping, especially since this has been done before. There are many papers doing exactly that, all of them extensively referenced in our manuscript. The scope of Nature Communications is to show impactful findings in a concise manner, which is what we're doing. Full length detailed descriptions of Maoniuping are a better fit for journals such as Journal of Petrology (as has indeed been done, more than once). Therefore, in order to stay within reasonable length limits and scope of our current target journal, we will only add information when it is absolutely required for the understanding of our suggested process.

The temporal and spatial relationships of the carbonatite magma, brine-melt and hydrothermal fluids at Maoniuping need clarification first and foremost.

This is already clear- a carbonatite melt evolves to brine melt by (1) crystallising calcite, and (2) reacting Ca and Mg away to silicates. Hydrothermal fluids coexist with the various melts at all stages. This process is present several times in the manuscript and we are not sure what additional clarification is required. Specific critique will be useful.

It is often not clear what rock unit the authors discuss about.

Line 131: Carbonatite only occurs at a very small outcrop in the Guangdoushan open pit. The authors need to note the location of samples in the figures of this study. Throughout the context, the authors should not generalise their work to the entire Maoniuping, because it contains two open pits which have varied geology, and the mineralisation style varies at depth.

You are correct, this information has been overlooked in the paper. All samples and photos are from Dagudao, not from Guangdoushan. The Guangdoushan part of Maoniuping is poorly mineralised, and accordingly consisting not many antiskarns. This is

in the manuscript now and serves as additional indirect evidence for the importance of antiskarn reactions to the Maoniuping REE mineralisation.

Line 131-132: What is the spatial relationship of the carbonatite and ore vein? And what are their mineralogical differences/similarities?

The difference between "carbonatite" and "ore vein" is already clearly explained via the modal abundance of calcite in the following paragraph.

Line 134: The Maoniuping seems to have > 20 ore veins in contact with syenite (and granite). Figure 2a-d can hardly explain the ubiquities of pyroxene of aegirine-augite and aegirine. Can you provide an estimate of the ubiquities of the occurrence?

Maoniuping has many more than 20 ore veins, actually closer to several hundreds. This is a very common texture in Dagudao section of Maoniuping, as shown by many other studies on Maoniuping before. Some examples from three different groups are Liu et al 2019 MinDep, Weng et al 2022 OGR, Xie et al 2009 OGR. All these and many more are already cited in the paper where appropriate. A quantitative or semi-quantitative survey of how many of the veins have antiskarns in them is beyond the scope of this manuscript, but it is the majority of them at Dagudao.

Furthermore, Fig. 2a actually shows the pyroxene develops at the carbonatite-syenite contact, instead of the vein-syenite contact. For the real ore vein-syenite contact, the pyroxene zone is either not obvious or missing in Figs. 2b and 2c.

As above, the difference between "ore vein" and "carbonatite" is mostly up to the relative modal proportion of calcite. Both "ore veins" and "carbonatites" are cumulate rocks crystallising from a carbonatite melt or brine-melt, and as such both can react with the silicate rocks to form antiskarns. This is now clarified in the manuscript.

Line 137-139: Detailed field photos or thin section micrographs need to be provided to show the growth direction.

Clarified that the pyroxene zones are forming outwards. Not necessarily that pyroxene has any preferred orientation (as it probably does not). Note that the alternative hypothesis, that pyroxene somehow nucleated several cm away from syenite and then grew towards it is unrealistic and would need quite some petrological gymnastics to explain.

Line 158: The authors also make this "ore vein-carbonatite" distinction in their figures and text. They should clarify what they define as "ore veins" before demonstrating the textural relationships in the figures.

The very same line you're quoting here is the one that defines what ore veins are and how they are distinct from carbonatites—we do not understand this comment.

Line 163-163: Please add thin section petrography work as supplement to support this statement. Hardly see mineral textures from Fig.2.

Figure 2j shows this very well, we do not understand how it is "hardly". Also added a reference to figure 4b which already shows exactly what you are looking for.

Line 166-168: Crystal shapes cannot be determined from these photos.

Maybe you had a low resolution version of the figures? Fig 2f clearly shows fluorite cubes and calcite rosettes. 2e shows prismatic bastnasite very well. Amphibole needles are obvious in almost all figure panels. 2c shows tabular K-feldspar.

(6) Figures are poorly illustrated or organised. Suggestions for improvement are listed below.

Figure 1: Define where and which open pits these outcrops are located.

Done–Dagudao. Coordinates and direction added.

Figure 1b: Quantify how common this is? And do the ore veins also contain cpx? Or only around the edges? What are the spatial relationships of the pyroxene band with the ore veins? Are the ore veins always associated with a pyroxene band?

Answers to all these questions were either already answered in the paper, or added to the present revision.

Figure 2: This figure is used as evidence that pyroxene grows outwards (within the carbonatite conduit), a feature which is not well visible from this figure. The authors need to provide more detailed images.

Again, I suspect that you had low resolution figures. Figures 2a and 2d clearly show angular syenite xenoliths with pyroxene mantles, within the carbonatite or ore vein matrix. There is no question about it. Once you see that and know how to recognise it, then it is very well visible in 2b and 2c as well. What exactly are you looking to see in "more detailed images" that isn't already visible here?

Add a table of the mineralogical composition and proportions of the different units.

Which "units" and what mineralogical composition are you looking for? If this is the distinction between stockworks, veinlets, and coarse veins, then we now added it to the revised manuscript. Which minerals? What remaining questions do you have regarding the model we are presenting in our manuscript, that mineralogical compositions that aren't already in the manuscript are required for?

Figure 2: In Fig. 2a, pyroxene-amphibole band forms, but there seems to be no REE mineralisation in the calcite carbonatite. Whereas in Fig. 2d there is REE mineralisation in the so called 'ore vein' in association with pyroxene band. What causes this difference?

Good observation and excellent question. First of all, in all cases, there will be REE mineralisation very close (centimetres) to an antiskarn. This is obvious in many figures, although admittedly not all of them. As for the actual observation of spatial separation, we do not have a clear answer for it, but two guesses: (1) The calcite-rich part formed at somewhat higher temperatures before the local melt differentiated sufficiently to form a proper brine-melt, or (2) this is adjacent to an ore vein, not visible at this photo scale. Brine-melts are excellent diffusion mediums, and it could be that ore minerals nucleated in one spot, literally tens of cm away, depleting this particular zone from ore elements resulting in only calcite. This is a common feature of carbonatites.

However, we choose not to explore these possibilities because, again, there is no good answer and any speculation will not benefit the manuscript.

Figure 3: Provide a thin section to show the transition from antiskarn to Ca-rich carbonatite.

The transition from pyroxene to calcite is rather abrupt, as is clearly evident in Fig 2, and in many examples in previous papers, all of which are cited here.

A gradual change would be expected if the antiskarn formed from the carbonatite melt.

Not at all. Experimental evidence shows the transition to be abrupt, and antiskarn studies elsewhere in the world very often show this transition to be abrupt and sharp.

What does white represent in figure 3b and 3c?

Epoxy resin – now added to the caption.

There is a yellow rectangle on the right bottom in figure 3c- delete it.

This yellow rectangle serves a purpose, and it is already explained in the figure caption.

Rotate either fig. 3a or 3b or 3c or make the orientation aligned.

This is not possible because 3b and 3b are different orientations with respect to each other. There is no one single rotation that can correctly align all panels of figure 3. This is also not necessary, as we believe most readers will be able to understand the orientation on their own.

Figure 4: Please add a scan of the thin section either in figure 4 or as a supplement, which shows the co-occurrence relationship of REE minerals and those silicate minerals being measured.

Figure 3 is literally this – just showing a mineral map instead of a PPL/XPL thin section, arguably a superior method to identify minerals. This is in the caption of both figures 3 and 4.

Please consider adding electron probe data for a line across the pyroxene crystal in the elemental maps, to have more quantitative assurance of the Na/K/Fe/Mg variation (line 184).

Good suggestion – quantitative data now added to the figure, with actual data in the supplementary data tables.

Need to re-organise the order of plates. Fig. 4g goes before fig. 4c.

Why? Is there a rule that BSE images must precede WDS maps? Please explain the rationale for this request.

Also need to state where this sample is from, i.e. ore vein.

Done

Be consistent with naming cpx or aeg-aug in the plates.

Done

What colour scale for the WDS elemental maps? Pinkish = higher concentration?

Yes – colour scale now added to the figure.

Figure 5: Please list all the elements on the X-axis.

All elements will not fit, but added Ce and Nd.

The REE element pattern is more like a humped sinusoidal pattern, instead of sinusoidal.

This is a very unimportant distinction.

Also note that in reference 54, the REE element pattern in fig.6 is primitive mantle normalised, while your data is Chondrite normalised.

This does not matter because the primitive mantle is essentially ×10 chondrite, equally for all elements. All it does it shift the pattern up or down, and does not affect shape or curvature at all.

Fig. 5a: Where are these samples from? What mineral assemblage?

These are the same samples from the rest of the manuscript, with the same mineral assemblages. Note added to figure caption.

Add literature data on Cpx-Amp REE patterns from carbonatite and non-carbonatite (i.e. silicate) magmas.

Literature data on cpx-amp ree patterns already exists in ref 54 (Reguir et al), which you yourself just mentioned two sentences above. REE patterns from silicates are very variable, but typically HREE-enriched for cpx, and MREE-enriched for amph. This is now added to the manuscript. It is difficult to add a citation, because unlike for carbonatites, there is no review paper about "REE patterns of diopside in silicate rocks".

Fig. 5b: Where do these fluorite-calcite pairs originate from? What rock, lithological unit, location? Please consider providing a thin section to show the mineral assemblage and the textural relationship to demonstrate the co-genetic relationship between fluorite and calcite. Hard to judge what this data represents.

This data represents magmatic co-crystallisation of calcite and fluorite from a carbonatite melt, in equilibrium. There is simply no other way for their REE ratios to exactly reproduce experimental partitioning ratios. This is now further explained in the manuscript. Data provenance added to supplementary information. We also added additional calcite–fluorite pair data from Liu et al 2019 to further show this is prevalent throughout Maoniuping, and not just a one-off.

The authors also need to explain more explicitly in the context what element activities might have caused the order of difference (line 209) and how the experimental set-up compares to Maoniuping.

Without going too much into crystal chemistry and partitioning theory, those are probably activities of monovalent elements (Na, K, etc) that can charge balance the trivalent REE when they're substituting divalent Ca in fluorite and calcite. This is now clarified in the manuscript. The experimental setup does not matter – experiments are used to derive equilibrium thermodynamics which are then applicable universally and need not replicate Maoniuping or any other natural system.

Figure 6: This figure aims to illustrate a new crystallisation process. However, the metasomatic freezing mechanism involves the removal of alkalis by the introduction of new components into the system (predominantly Si). As the composition of the melt is externally changed, this defies the point of using a eutectic (homogeneous) system to explain the process? This is not a simple crystallisation process; it involves changing the melt composition through metasomatism. I don't think you can visualise this on

such a binary phase diagram. The authors can consider using a different figure, maybe experimental figures would be better, to show how the temperature remains stable while antiskarns are forming.

This figure aims to illustrate the process via comparison to previously known processes. Yes, exactly, the composition changes. It doesn't mean the system stops being eutectic. It is still very much eutectic, but the eutectic composition is never reached. The lack of fluxing elements (Na and K in this case) push the composition sideways. We think it is a very good way (albeit admittedly simplified) to visualise the process. I am not sure which "experimental figures" the reviewer is referring to since no experiments were conducted in this study. Nonetheless, this has been clarified in the figure caption. Mentions of "isothermal" and "freezing" removed.

Other minor comments are listed below and added in the annotated manuscript. A couple of places are missing key references and flagged out in the annotated manuscript.

These issues are addressed separately in the attached manuscript file.

Title: The title doesn't reflect the discussion points of this manuscript. Either magma solidification or reaction freezing is sufficiently explained in the context.

Title revised.

Abstract: Add how REE mineralisation is related to the antiskarn formation and wall rock contamination.

Not much leeway because of word limit, but some modifications have been made.

Introduction: Need more introduction on magmatic metasomatism, antiskarn and the genetic relationship of carbonatite magma, brine-melt and hydrothermal fluids in carbonatite REE system in general.

We believe it is already well introduced. If there are specific questions the reviewer has, we will be happy to address them.

Line 11: The authors are mixing things up. Magmas do not solidify as a result of degassing. The solidification process is only temperature driven. The volatile degassing is an effect of exsolution by decompression.

We are not mixing things up. Decompression -> exsolution and loss of H_2O -> higher melting point -> solidification.

Line 12 - 13: Not accurate. Magmatic metasomatism involves a silicate melt phase reacting with host rock. It is not only about H2O: see page 282-283: https://link.springer.com/article/10.1007/BF00307287

We did not say it is *only* H2O. You are correct, but magmatic metasomatism is overwhelmingly understood to form via expelled fluids. This is an abstract and there is no need to explain every single exception to the rule.

H2O is emphasised here for its role in forming metasomatic fluids, but in the later discussion section, H2O is barely discussed but instead SO2 and CO2 were discussed.

Exactly – this is the point. We are showing a metasomatic process that is not H_2O-driven. Note that our mentions of CO_2 and SO_2 were referring to things that are happening during antiskarn formation, but are not crucial to the process. However, their presence

provides additional evidence for antiskarnisation. Hence, it is magmatic, not hydrothermal. This is already clear enough in the manuscript and no revisions made.

Line 26-27: I don't think this manuscript has provided sufficient evidence to conclude the relative importance of wall rock contamination and T decrease/volatile degassing. Modelling may provide some insight.

We disagree. The antiskarns are chock full of aegirine, arfvedsonite, phlogopite. You simply cannot make them by cooling or degassing. What would be sufficient evidence? Which kind of modelling is the reviewer expecting?

Line 34-35: This is not a description of process. This is a description of fact (if that is true).

We don't understand this comment. The next sentences describe the process. What is the issue?

Line 41: What about FeCO3? Since ankerite is mentioned in Line 44.

It is enriched, forming ankerite. References cited in that area describe it in more detail, which is not crucial for the paper's introduction.

Line 66: It is an odd turning. The end of last paragraph presents a controversy on whether the ore-host rock is a carbonatite or hydrothermal rock. The following paragraph then starts with 'as with most carbonatite..' Abundant fluid inclusions can also exist in hydrothermal rocks. Don't misguide readers.

No one is misguiding anyone. This is clearly spelled out in the paper: Maoniuping is a carbonatite. Previous work suggested that the mineralisation is hydrothermal, but that does not mean Maoniuping is not a carbonatite. This is already clear enough in the paper, and the whole paragraph starting in line 66 talks exactly about that. Additionally, isotope studies conclusively demonstrated that the ore-veins are isotopically identical to the Maoniuping magmatic carbonatites. This information is now added to the manuscript.

Results:

Line 179: 'Commonly' is overstated. Only one amphibole crystal was measured by EPMA spot, and shows difference in chemical composition. In the WDS element maps, the two Amp crystals show moderate enrichment of Ca at the rim, but for other elements, the zoning is not obvious.

There is obvious zoning in the two Amp crystals, with Ca decreasing and Na increasing towards the rim. This is exactly what we're claiming. We never said anything about the other elements, so the fact that they're "not obvious" is not an issue. The EPMA spots show clear zoning. What is overstated about "commonly"? How many amphibole BSE images with line profiles along them are required to convince the reviewer that amphiboles are commonly zoned?

Line 184: Please consider adding electron probe data for a line across the pyroxene crystal in the elemental maps, to have more quantitative assurance.

Compositions (as Na/Ca) added, with full numbers in the supplementary tables. That said, we believe the zoning is very obvious and there is enough assurance that this is indeed the case. We also point out that the TIMA maps show exactly this type of zoning, across many *hundreds* of crystals.

Discussion:

Please provide decent petrographic evidence to show the minerals growth pattern and paragenetic sequence, especially the growth direction of pyroxene.

This was addressed in an earlier comment.

Contradicting points were made in terms of the paragenesis. The authors suggest 'amphibole and phlogopite most likely formed at the same time (line 247)', which seems to contradict with line 163: "Within veins, amphibole formed first.."

Correct, we removed the mention of phlogopite.

The authors will need to present a mass balance estimate to support the theory based on reaction 2 (line 262-265). The pyroxene reaction zone doesn't seem to be prevalent geologically and cannot consume a huge amount of Fe to explain the lack of magnetite.

The pyroxene zone is very prevalent geologically. Figure 1a, for example, shows a metre-scale chunk of pyroxene. This alone is sufficient to equivalent to many metres cubed of magnetite-bearing calcite carbonatite. Remember that whilst magnetite is common in carbonatites, it is only really common in early calcite carbonatites and much rarer in evolved brine-melt-derived carbonatites. So not much is expected anyway. This is now clarified in the manuscript.

Additionally, what is stated in this section is that Fe^{3+} is generated by redox coupling CO_2–H_2O and Fe^{2+}. How does one convincingly conduct a mass balance for this? One does not – it is a poorly constrained game of fudging numbers and an astute reader will not fall for it. Might as well not even try.

The authors will also need to provide a larger area of micro-XRF scan than figure 3 to demonstrate a transitional zone from the pyroxene reaction zone to calcite carbonate (or calcite abundant area in the ore vein). The petrographic evidence will be essential to support the reaction series and conclusions in line 272-274.

There is no transitional zone, as described above, and there is no expectation for such a zone. The conclusions in line 272 (perthites) are now supported by an additional figure showing mineral maps (done by TIMA, not XRF as claimed by the reviewer)

What is the discussion on feldspar all about? To suggest their formation in a hot environment?

Based on A&W2024, feldspar perthite can be used as a temperature constraint, which is exactly what we're doing.

And what does it have to do with the antiskarn formation?

Feldspar is part of the antiskarn. It is a silicate that can only form via contamination. This is clarified in the manuscript.

If the authors want to use the feldspar discussion to support their point, they need to provide their own petrographic evidence of co-genesis of the two distinct feldspar and their co-genesis with the REE minerals.

Petrographic evidence added, there is now a whole figure showing exactly this.

Reviewers’ comments below in dark blue, with our responses in orange.

Figure 6a is not in its appropriate use. Consider deleting it or use another phase diagram. The binary eutectic phase diagram is normally used for an iso-chemical system. It is okay to use the diagram to describe the change of carbonatite melt from A to B, but not from B to C. During this stage, according to the authors, alkalis are removed from the carbonatite melt or brine-melt through reactions with the host rock, a process that is accompanied by the incorporation of Si and Al (± K) into the melt. This is no longer an iso-chemical system, nor a pure magmatic system, and the temperature doesn’t stay the same.

From B to C, the diagram gives the illusion that the (Na,K)2CO3 component of the initial melt is removed, hence enriching the fluid in the (Ca,Mg)CO3 component. The only way to achieve that in such a binary eutectic diagram is through fractional crystallisation of pure Na-K carbonates. But that is not what is happening. In fact, only the alkalis are removed, and not in the form of (Na,K)2CO3 as the diagram suggests. Instead, the alkalis are sequestered in silicates. The externally added silica and aluminium were not part of the starting composition, and do not fit anywhere in this two-component carbonatite melt system. In other words, from A to B occurs within a closed carbonatite melt system (two-component), whereas the transition from B to C takes place in an open and reactive (multi-component) magmatic-metasomatic system. Hence, this not a suitable figure to visualize this process.

We see your point of view and indeed this is a very qualitative and unconstrained pseudobinary. Given that it’s not too important anyway, we adopt your recommendation to delete it from the paper, and update the text accordingly.

Line 39: What do you mean by ‘proper’ hydrothermal fluids?

Proper meaning aqueous – clarified in the manuscript.

Line 123: ‘’a purely magmatic model’’. The model that the authors propose lacks hydrous REE-enriched fluids, but is definitely not purely magmatic. It involves solid-melt element exchanges, i.e. contaminating the melt through metasomatism.

It is magmatic in the sense that the minerals are crystallising from the magma. Metasomatism may not be the best word because metasomatism typically means replacement, but here we have a coupled non-hydrothermal replacement (fenite) and magmatic (antiskarn). In any case, for the sake of accuracy, we adopt your recommendation and remove the word “purely”.

Line 197: However, the discussion on LREE partitioning in lines 267-270 seems to assume calcite-fluorite pair crystalises earlier than the fluorite-bastnasite pair.

Yes, but this part in line 197 is textural. The fact that mineral chemistry shows continuous crystallisation over an interval is somewhat expected (and noted in previous studies as already appropriately referenced in our work). This line is now clarified that we’re talking about textures.

Line 279-281: Awkward sentence- rephrase.

Good catch—now corrected.

Line 290: K-feldspar is also consumed.

Yes, corrected.

Line 338: In addition to silica, aluminium (and possibly K) is also involved in the exchange.

Yes, but those aren't a required part of the antiskarn process. Silica transfer is the overwhelming driving force. No changes made.

Line 371: Give the quartz context. Quartz is not mentioned to the petrography of either anti-skarn or the vein interiors. Are the studied quartz with melt inclusions associated with late hydrothermal phases?

Yes, this is now added to the petrography section.

Line 385: ''a non-isothermal environment'' – but this is not what B to C in figure 6b shows.

Figure 6 removed, so not an issue.

Line 494: Move the sentence ''Elsewhere, bastnäsite … in burbankite'' to before line 492 (''At Maoniuping..''). First describe how bastnäsite typically forms, then discuss why this can't be the case for Maoniuping.

Done.

Line 533: It is not a frozen but a contaminated brine-melt.

Added "contaminated" for clarification and accuracy, but kept "frozen" because it conveys our point very well.

Line 565: It is a local phenomenon at Maoniuping.

Yes, we're talking only about the REE deposit, not the entire carbonatite body. This is already written in the paper. No changes made.
